# *Drosophila* Genome-Wide RNAi Screen Identifies Multiple Regulators of HIF–Dependent Transcription in Hypoxia

**DOI:** 10.1371/journal.pgen.1000994

**Published:** 2010-06-24

**Authors:** Andrés Dekanty, Nuria M. Romero, Agustina P. Bertolin, María G. Thomas, Claudia C. Leishman, Joel I. Perez-Perri, Graciela L. Boccaccio, Pablo Wappner

**Affiliations:** 1 Instituto Leloir, Universidad de Buenos Aires, Buenos Aires, Argentina; 2 Departamento de Fisiología, Biología Molecular, y Celular, Facultad de Ciencias Exactas y Naturales, Universidad de Buenos Aires, Buenos Aires, Argentina; 3 Consejo Nacional de Investigaciones Científicas y Técnicas, Buenos Aires, Argentina; University of California San Francisco, United States of America

## Abstract

Hypoxia-inducible factors (HIFs) are a family of evolutionary conserved alpha-beta heterodimeric transcription factors that induce a wide range of genes in response to low oxygen tension. Molecular mechanisms that mediate oxygen-dependent HIF regulation operate at the level of the alpha subunit, controlling protein stability, subcellular localization, and transcriptional coactivator recruitment. We have conducted an unbiased genome-wide RNA interference (RNAi) screen in *Drosophila* cells aimed to the identification of genes required for HIF activity. After 3 rounds of selection, 30 genes emerged as critical HIF regulators in hypoxia, most of which had not been previously associated with HIF biology. The list of genes includes components of chromatin remodeling complexes, transcription elongation factors, and translational regulators. One remarkable hit was the *argonaute 1* (*ago1*) gene, a central element of the microRNA (miRNA) translational silencing machinery. Further studies confirmed the physiological role of the miRNA machinery in HIF–dependent transcription. This study reveals the occurrence of novel mechanisms of HIF regulation, which might contribute to developing novel strategies for therapeutic intervention of HIF–related pathologies, including heart attack, cancer, and stroke.

## Author Summary

Adaptation of cells to low oxygen (hypoxia) is a physiological response related to important diseases, including heart attacks, stroke, cancer, and diabetes. The mechanisms that mediate adaptation to hypoxia in humans are almost identical to those operating in diverse animal species, including mice, worms, and insects. The master regulator of cellular responses to hypoxia is a transcription factor named HIF, which induces a set of genes that mediate adaptation to oxygen starvation. Although it is known that regulation of HIF occurs mainly at the level of protein degradation and transcriptional coactivator recruitment, a comprehensive screen for HIF regulators has not been performed before. In this work, we have conducted an RNAi-based screen of the genome of the fruit fly *Drosophila melanogaster*, searching for genes that are required for HIF activity. This screen carried out in a cell culture system led to the definition of 30 critical regulators of HIF, most of which have not been associated with hypoxia biology before. The hits of the screen included components of chromatin remodeling complexes, transcription elongation factors, and translational regulators. Our results open the possibility of performing detailed studies on HIF regulation that may lead to novel therapeutic strategies for important human diseases.

## Introduction

The cellular response to low oxygen tension (hypoxia) involves changes in gene expression that mediate adaptation to this condition. The hypoxic response is primarily mediated by a family of highly conserved transcription factors named Hypoxia Inducible Factors (HIFs) [1]. HIFs are α/β heterodimers, in which the common β subunit is constitutive and α subunits are negatively regulated by O_2_ through several concurrent mechanisms that include oxygen-dependent proteasomal degradation [2], blockage of transcriptional co-activator recruitment [3], [4] and subcellular localization [5], [6]. HIFα proteolysis requires polyubiquitination, which in turn depends on the hydroxylation of two key prolyl residues localized in the so-called oxygen-dependent degradation domain (ODDD) [7], [8]. Hydroxylation is mediated by specific HIF prolyl-4-hydroxylases, named PHDs that utilize dioxygen as a co-substrate, and hence, are considered *bonafide* cellular oxygen sensors [9], [10].

The machinery that mediates the transcriptional response to hypoxia is conserved in *Drosophila melanogaster* [11], being Sima and Tango the fly orthologues of HIFα and HIFβ [12] respectively, and Fatiga, the single *Drosophila* PHD [13]. As in mammalian cells, Sima is stable in hypoxia but rapidly degraded in normoxic conditions; its degradation requires Fatiga-dependent hydroxylation of a specific prolyl residue localized in the Sima ODDD [12], [14]. The *fatiga* gene is in turn transcriptionally activated by HIF, defining a negative feedback loop [12], [15].

HIF plays a crucial role in several human pathologies, including coronary heart disease, stroke and cancer [16], [17], and thus, considerable effort has been devoted to the characterization of the cellular response to hypoxia, and to the identification of HIF regulators that may contribute to developing novel strategies for therapeutic intervention. Various small molecule screens searching for HIF regulators have been conducted using high-throughput approaches (see [18] for a review). Although these strategies have been instrumental for manipulating HIF-dependent transcription, they have resulted less informative for the identification of the molecular targets involved.

In this work, we have carried out a genome-wide RNAi screen in *Drosophila* Schneider (S2) cells, aimed to the identification of genes required for HIF activity in hypoxic conditions. We have identified 30 regulators of the HIF response, including some previously reported genes, such as members of the phosphoinositide 3-kinase (PI3K) and Target of Rapamycin (TOR) signaling pathways [19], subunits of the COP9 signalosome complex [20], [21], and components of the Brahma chromatin-remodeling complex [22]. Among the genes identified as novel regulators of HIF-dependent transcription, we found the chromatin modifying elements Reptin and Pontin, several transcriptional and translational regulators, and the miRNA pathway component Argonaute 1. Further analysis confirmed an absolute requirement of core components of the miRNA machinery for the hypoxic response, both in cell culture and *in vivo*, suggesting a physiological role of miRNAs in HIF activity.

## Results/Discussion

### Genome-wide RNAi screen for HIF regulators

**Figure 1:**
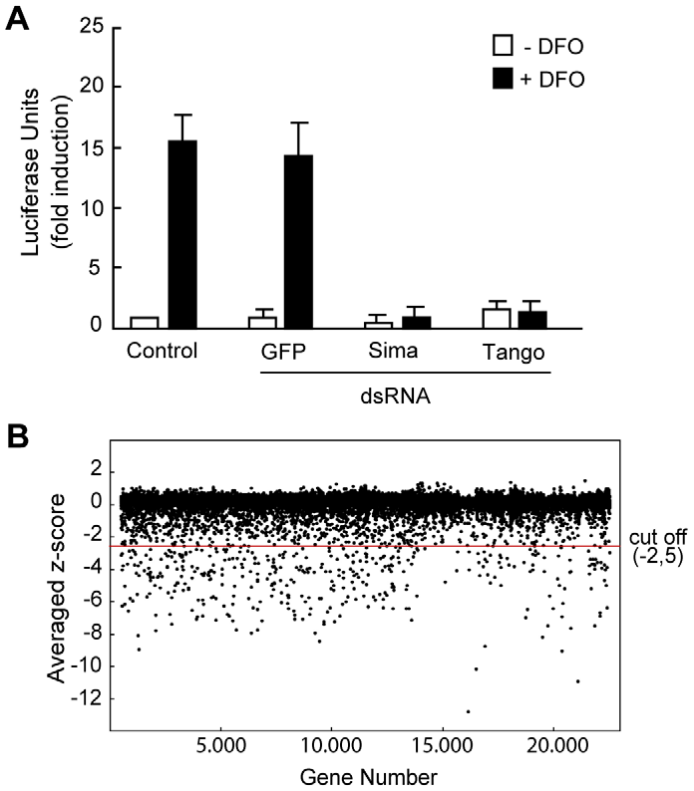
Primary screen for genes required for HIF–dependent transcription. (A) S2-HRE-Luc cells treated with dsRNA against *gfp* (negative control), *sima* or *tango* were exposed or not to DFO. Luciferase induction by DFO was abrogated in cells depleted from *sima* or *tango*. (B) Scatter plot of the average Z-score (see Materials and Methods) of the whole set of data of the primary screen. dsRNAs which reduced reporter gene expression with a Z-score of less than −2.5 (cut-off line) were selected as positive hits of the primary screen for further analysis.

The genomic screen was carried out in *Drosophila* S2 cells bearing a stably-transfected hypoxia inducible reporter, in which a HIF-Responsive-Element (HRE) derived from the murine *lactate dehydrogenase-A* (*ldh-A*) enhancer drives the expression of *firefly* luciferase (Figure S1A; [15]). The HRE-Luc reporter was strongly induced upon exposure of the cells to hypoxia or to the iron chelating agent desferrioxamine (DFO), a compound that mimics the effect of hypoxia (Figure S1B) [15]. RNAi pilot experiments demonstrated that induction of the HRE-Luc reporter was dependent on Sima and Tango (Figure 1A) [15], and therefore, served as a reliable assay for testing the genomic double stranded RNA (dsRNA) library of the RNAi Screening Center (DRSC; http://flyrnai.org) that corresponds to more than 90% of the *Drosophila* transcriptome [23].

The screen was divided in 3 sequential phases (Table S1; see also Materials and Methods): I) a primary screen carried out in cells exposed to DFO, using a first-generation genomic library (DRSC 1.0 library) [23]; II) a secondary screen in which the genes that scored as positives in the primary screen were re-tested in cells also exposed to DFO, using a second generation library (DRSC Validation library) [24], [25], and normalizing the results with a constitutive transcriptional reporter (see below); and finally, III) a tertiary screen in which genes that scored as positives in the two previous phases were tested in hypoxia (1% O_2_).

I) The results of the primary screen were highly reproducible with Z score values (see Materials and Methods) showing a correlation coefficient of 0.6 between duplications (Figure S1C). A few dsRNAs rendered less reproducible results (i.e. the duplicates were more divergent), but nevertheless, were included in the secondary screen to avoid loosing potentially relevant hits. As shown in Figure 1B, approximately 97% of the dsRNAs rendered Z score values of around zero, indicating that, as expected, the majority of them do not affect HIF-dependent transcription. The screen was carried out in cells exposed to DFO and therefore, set up for the identification of positive regulators only. Thus, a substantial number of genes rendered negative Z score values (putative activators) but no genes with significant positive Z-score values (putative inhibitors) were obtained. We decided to define a Z score cut-off value of −2.5 for a gene to be considered a hit of the screen (Figure 1B) and, based on this criterion, 603 genes were initially selected for further analysis (Table S2). Noteworthy, both *sima* and *tango* -the *Drosophila* HIF-alpha and beta subunits respectively- scored as positives in this primary selection, with Z scores of −6.4 and −4.1 respectively, suggesting that the screen is reliable and has the potential to identify novel genes required for HRE-dependent transcription. Next, in order to eliminate genes that presumably interfere with basic cellular functions and prevent cell viability, the 603 hits were filtered against the results of a RNAi genome-wide screen for genes required for cell viability, previously carried out in the same cellular system with the same library [23]; 311 genes fell in the “cell viability” category, so they were not pursued further. Open reading frames that have been predicted but never demonstrated (the “Sanger collection”: 67 genes) were also eliminated from the analysis. Thus, after filtration, the number of positive genes from the primary screen was reduced to 225 (Table S3).

II) For the secondary screen, we developed a stably transfected cell line, which contained, along with the HRE-*firefly* luciferase reporter, a constitutive actin-*Renilla*-luciferase element, which was used to normalize the results (see Materials and Methods). This phase of the screen was carried out with a second-generation library (DRSC Validation Library; http://flyrnai.org), which was designed to eliminate false positives that arise from off-target effects of the original library [24], [25]; this new library includes more than one dsRNA for most genes (Table S4). Like in the primary screen, DFO was used as the hypoxic-mimetic agent (Table S1).

At the secondary screen, those genes that provoked a reduction of HRE-Luc reporter activity of more than 50% with at least one of the two dsRNAs were considered as hits. On this basis, 66 genes scored as positives, and based on the strength of the effect, this set of genes was further classified into two categories: Group A) Genes that rendered -with at least one of the corresponding dsRNAs- over 75% inhibition of HRE-*luciferase* activity (23 genes), and group B) Genes that provoked an inhibition of 50–75% of the activity -with at least one of the corresponding dsRNAs- (43 genes) (Table S4). As expected, *sima* and *tango* were among the hits of the secondary screen with approximately 96% inhibition.

III) Finally, we carried out a tertiary screen, in which genes that scored as positives in the secondary screen were tested in cells exposed to hypoxia (1% O_2_). All 23 genes that scored in group A (strong inhibition) were included in the tertiary screen, along with a selected set of genes from group B (12 genes) that are functionally related to those from group A. Thus, a total number of 35 genes were analyzed in hypoxia (Table 1). In this final screen 30 genes, including *sima* and *tango*, scored as positives with at least one of the two dsRNAs provoking more than 50% inhibition of HRE reporter activity (Table 1). Genes already known to be required for the HRE response, such as elements from the PI3K/TOR pathway [19] and the COP9 signalosome complex [20], [21], as well as genes that were not previously linked to HIF (see below), were among the hits in this final phase of the screen (Table 1).

**Table 1:** List of genes that scored as positives at the tertiary screen.

Grouping criteria	*Drosophila* gene	Human homologue	Known or inferred function	inhibition (%) amplicon 1	inhibition (%) amplicon 2
HIF	Sima	HIF alpha	Alpha subunit of HIF	92,3+/−6,4	89,3+/−5,4
	Tango	HiF beta/Arnt	Beta subunit of HIF	59,0+/−1,1	-
Protein translation	CG9769	eIF3f	Subunit-f of eukaryotic translation initiation factor 3 complex	91,9+/−3,7	-
	Tango7	eIF3m	Subunit-m of eukaryotic translation initiation factor 3 complex	87,4+/−2,8	61,7+/−25,7
	Trip1	eIF3i	Subunit-i of eukaryotic translation initiation factor 3 complex	87,5+/−3,4	84,7+/−11,8
	CG8636	eIF3g	Subunit-g of eukaryotic translation initiation factor 3 complex	85,1+/−10,2	-
	Pixie	RLI	Assembly of translation initiation complexes	74,5+/−7,9	77,7+/−14,2
	CG4849	eEF2	Putative translation elongation factor (downstream of TOR/S6K)	83,3+/−2,1	42,0+/−11
	Ef2b	eEF2	Translation elongation factor (downstream of TOR/S6K)	73,9+/−16,1	67,7+/−12
PI3K/TOR signaling	dTOR	TOR	Target of Rapamycin kinase	89,5+/−5,5	-
	dRaptor	Raptor	Component of TORC1 complex	89,3+/−2,3	66,9+/−3,1
	dRheb	Rheb	GTPase required for TOR activity	82,1+/−11,9	54,6+/−31,2
	dPDK1	PDK1	3-phosphoinositide-dependent protein kinase 1	56,2+/−8,3	51,5+/−24,5
Chromatin remodelling	Brahma	Brahma	ATPase component of the SWI/SNF complex	83,9+/−11,6	84,8+/−0,6
	Bap155/Moira	BAF170	Component of the SWI/SNF complex	77,9+/−5,3	77,5+/−2,3
	Snr1	Ini1	Component of the SWI/SNF complex	38,0+/−15,3	28,2+/−11,4
	Bap60	BAF60a	Component of the SWI/SNF complex	34,2+/−2,3	16,1+/−33.3
	Dalao	BAF57	Component of the SWI/SNF complex	35,3+/−7,9	-
	Reptin	Reptin	AAA+ ATPase component of various complexes	80,3+/−0,4	74,6+/−8,7
	Pontin	Pontin	AAA+ ATPase component of various complexes	75,3+/−4,6	67,3+/−17,5
mRNA processing	CG14641	RBM22	RNA binding motif protein 22 -Putative pre mRNA splicing factor	85,2+/−7,5	64,3+/−16,8
	Prp8	Prp8	RNAse H component of the spliceosome catalytic core	72,5+/−20,5	71,1+/−2,3
	Clipper	CPSF-30K	Subunit of Cleavage and Polyadenylation Specificity Factor	63,0+/−10,6	-
	Symplekin	Symplekin	Protein associated to mRNA polyadenylation complex	58,8+/−1,0	46,2+/−16,9
	Peanuts	-	ATP dependent RNA helicase involved in RNA splicing	31,2+/−41,3	-
microRNA	Argonaute 1	Ago proteins	Component of the miRNA pathway	88,4+/−8,1	69,7+/−9,7
Signalosome	CSN3	COPS3	COP9 complex subunit 3	60,3+/−2,6	30,5+/−3,7
	CSN6	COPS6	COP9 complex subunit 6	41,6+/−7,0	-
Miscellanea	Spt6	Spt6	Transcription elongation factor involved in heat shock response	88,9+/−2,7	75,2+/−0,1
	CG2446	-	Unknown	84,9+/−5,3	-
	TER94	p97	ER chaperone involved in ERAD	78,7+/−0,2	75,3+/−1,8
	Cryptocephal	ATF4	Transcription factor involved in stress responses	70,7+/−2,8	-
	MBD-R2	PHF20	Unknown function - DNA interacting protein	69,9+/−8,9	31,9+/−18,9
	CG7065	-	Unknown	64,8+/−19,6	-
	NSL1	-	tRNA aminoacylation	63,0+/−12	62,8+/−11,7

Cells were exposed to hypoxia, and dsRNAs corresponding to genes that provoked strong reduction of the response to DFO in the secondary screen (“Group A” genes) as well as some selected genes that rendered milder reduction of the response to DFO (“Group B” genes) were tested for their capacity to interfere with HRE-Luc reporter induction. Depicted genes are grouped according to their molecular function. Normalized luciferase activity (*firefly* to *renilla* luciferase activity ratio) for each well was calculated and expressed as the percentage of inhibition respect to hypoxic control cells treated with dsRNA against GFP. One or two amplicons (dsRNAs) were used for each gene. Amplicon identity is depicted in Table S4; their sequence can be found in http://flyrnai.org.

Four genes of the PI3K and TOR pathways -PDK1, TOR, Rheb and Raptor- were among the positive hits. Although it is still a matter of some controversy, several studies suggested that activation of PI3K/TOR pathway is required for HIF-dependent transcription [19], [26]. The fact that four elements belonging to these pathways were in the final list of hits strongly supports the notion that they are critically required for HIF activity.

One subunit of the eIF3 translation initiation complex, eIF3e/Int6, was previously shown to contribute to mammalian HIF-2α degradation [27]. In this screen, 4 additional subunits of this complex scored as positives as well (Table 1), implying that eIF3 complex involvement in HIF regulation might be broader than previously anticipated. The eIF3 complex is a scaffold for protein translation initiation composed of 12–13 polypeptides [28], and noteworthy, some of eIF3 subunits are associated to specific cellular events, such as oncogenic transformation [29] and apoptosis [30]. This work has now revealed that additional eIF3 subunits are required for HIF-dependent transcription. Several genes involved in chromatin remodeling, including 5 genes from the Brahma (also known as SWI/SNF) complex, and two from unrelated complexes -*pontin* and *reptin*- were also hits of the screen (Table 1). One previous report suggested a role of the SWI/SNF in the response to hypoxia [22], and a central role of chromatin remodeling in HIF-dependent gene expression is increasingly evident [31]. Therefore, the current screen, along with previous reports, strengthens the notion that an array of chromatin remodeling factors contribute to HIF-dependent transcriptional responses to hypoxia. *Drosophila* Pontin and Reptin are closely related members of the highly conserved AAA+ family of DNA helicases, which, besides participating in chromatin remodeling, are involved in responses to DNA double-strand breaks and transcriptional regulation mediated by β-catenin, E2F1 or c-Myc [32] [33]. The precise role of Pontin and Reptin in HIF-dependent responses needs to be investigated in detail.

A transcription elongation factor, *Spt6*, which had not been linked before to HIF regulation, was also identified in the screen (Table 1). Spt6 is known to co-localize with the phosphorylated (active) form of RNA polymerase II in areas of active transcription, particularly during induction of stress-related genes [34]. Spt6 is recruited to heat-shock (HS) dependent promoters upon the HS stimulus; recruitment occurs within 2 minutes after the HS and depends on the Heat Shock Factor (HSF) [35], [36]. Our results therefore expand the notion that Spt6 is a component of transcriptional responses to stress, including now the cellular response to hypoxia.

The *Drosophila ATF4* homologue *cryptocephal* was another remarkable hit of the screen (Table 1). ATF4 is a bZIP transcription factor expressed at high levels in hypoxic areas of human cervix, brain, breast and skin tumors [37], and considered a central component of cellular responses to different types of stress, including the unfolded protein response (UPR), amino acid deprivation, oxidative stress and hypoxia. In hypoxia, PERK, an endoplasmic reticulum (ER) transmembrane protein kinase, is activated, leading to general inhibition of protein synthesis, thereby allowing upregulated translation of selective proteins including ATF4. As a result, ATF4 induces the expression of genes in response to hypoxia, but remarkably, this response is HIF-independent [19], [38]. Our data now suggest that ATF4 is required for HIF activity, adding a new layer of complexity to the mechanisms involved in the cellular response to hypoxia.

### Argonaute 1 and the miRNA machinery are necessary for the transcriptional response to hypoxia

**Figure 2:**
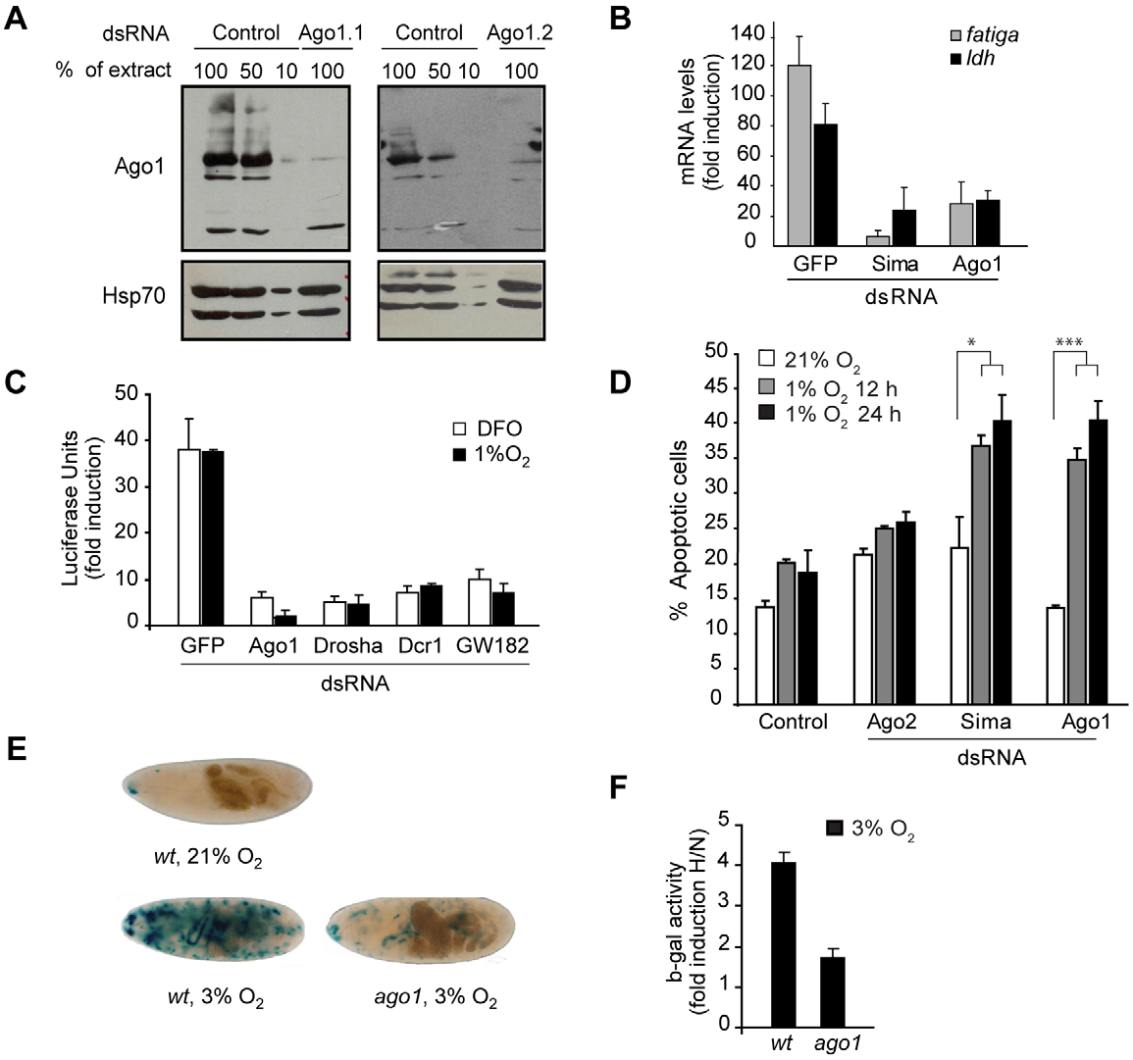
Argonaute 1 (Ago1) and the miRNA machinery are necessary for adaptation to hypoxia. (A) Western blot showing Ago1 strong reduction in cells treated with dsRNA against *ago1* during 4 days. Two different dsRNAs, *ago 1.1* and *ago 1.2* were used with identical results. Extracts from control cells were loaded at different amounts. Remaining Ago1 protein levels were 10% relative to controls after 4 days of RNAi treatment. Hsp70 was used as a loading control. (B) mRNA levels of two different HIF target genes, *fatiga* and *ldh*, were analyzed by real time PCR in cells exposed to hypoxia (1% O_2_) for 16 hours in comparison to those of cells maintained in normoxia. *sima* or *ago1* dsRNAs largely prevented hypoxic induction of *ldh* and *fatiga* transcripts. (C) S2-HRE-Luc cells were treated with dsRNA against *gfp*, *ago1*, *dicer-1*, *drosha* or *gw182* and then exposed to DFO or 1% O_2_. Whereas the *gfp* dsRNA had no effect on luciferase induction, silencing of any of the other genes strongly reduced luciferase induction by DFO or hypoxia. Data are represented as fold induction respect to control cells treated with dsRNA against *gfp*, and maintained in normoxia. (D) Analysis of the proportion of cells in apoptosis revealed that cells treated with *ago1* dsRNA were as sensitive to hypoxia as cells treated with *sima* dsRNA, whereas untreated cells or cells treated with *ago2* dsRNA were remarkably more resistant to low oxygen. After exposure to hypoxia, cells were stained with propidium iodide (PI) and Hoescht, and observed under a fluorescence microscope. The proportion of dying cells (PI positive) was determined using the CellProfiler cell image analysis software (Chi^2^ test *p<0.05; ***p<0.001). (E–F) Transgenic embryos bearing the hypoxia inducible reporter LDH-lacZ were exposed to hypoxia (3% O_2_) during 4 hours, and reporter gene activity was analyzed by X-gal staining (E) or quantitative β-galactosidase assays (F). The transgenic reporter is silent in normoxic wild type individuals, and strongly induced upon exposure to hypoxia (E). In *ago1^k08121^* homozygous mutants the expression of the reporter in hypoxia is clearly reduced (E–F; p<0.01). N = Normoxia; H = Hypoxia.

Argonaute 1 (Ago1), a central component of the microRNA silencing machinery [39] also scored as positive in the screen (Table 1). Given that little is known about the participation of the miRNA machinery in HIF regulation, we sought to further characterize Ago1 involvement in this process. We began by checking that dsRNA treatments were effective in reducing Ago1 protein levels (Figure 2A), and consistent with this, we confirmed that the function of the miRNA machinery was impaired (Figure S2A). To determine if inhibition of HRE-Luc reporter activity after Ago1 depletion reflects the behavior of endogenous hypoxia-inducible genes, we examined transcript levels of two well-established Sima downstream targets: *ldh* and *PHD*/*fatiga* [15]. The two transcripts were strongly upregulated in cells exposed to hypoxia, and this induction was dramatically reduced in cells treated with *ago1* dsRNA (Figure 2B). In order to assess if Ago1 is required in the hypoxic response as part of the miRNA pathway, we silenced other components of the miRNA machinery. dsRNAs for *dicer-1*, *drosha* or *gw182* strongly reduced luciferase reporter induction in cells exposed to DFO or hypoxia (Figure 2C), suggesting that the miRNA pathway is required for the transcriptional response to hypoxia. Other genes related to *ago1*, which have no reported function in the miRNA pathway, were also evaluated: Depletion of *argonaute2*, *piwi* or *dicer-2*, did not affect the HRE-response in S2 cells (Figure S2B). It is well known that HIF play a crucial role in the adaptive response to hypoxia by controlling the expression of genes that eventually promote cell survival. Thus, we studied if Ago1 does indeed contribute to cell viability in hypoxia. As depicted in Figure 2D, cells treated with *ago1* dsRNA and exposed to hypoxia enter apoptosis at a higher proportion than untreated cultures, or cells treated with control *ago2* dsRNA, suggesting a physiological requirement of Ago1 in the response to hypoxia. We next sought to test whether Ago1 is required for the HRE response *in vivo*. We analyzed the expression of the hypoxia-responsive transgenic reporter LDH-*Lac*Z [12] in *ago1^k08121^* mutant embryos. As previously reported, in wild type embryos LDH-*Lac*Z is silent in normoxia and induced in hypoxia in a characteristic expression pattern that corresponds to some of the developing tracheal branches [12] (Figure 2E). In contrast, in *ago1^k08121^* homozygous embryos, induction of the LDH-*Lac*Z reporter in hypoxia was much weaker, indicating that Ago1 contributes to HIF/Sima dependent transcription *in vivo* (Figure 2E and 2F).

**Figure 3:**
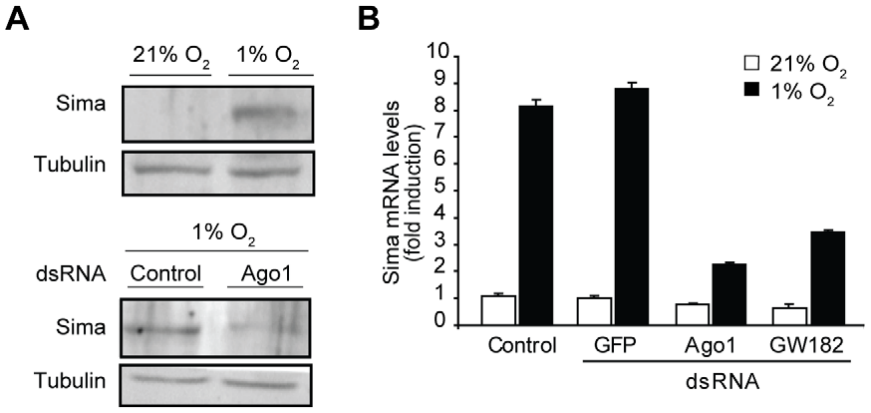
Hypoxic accumulation of Sima protein and mRNA is prevented in cells treated with *ago1* dsRNA. (A) Anti-Sima western blot analysis reveals that hypoxic accumulation of Sima is reduced in *ago1* RNAi treated cells (24 h at 1% O_2_). (B) Real time PCR revealed that *sima* mRNA is strongly induced in cells exposed to hypoxia, and this induction is largely prevented in cells treated with *ago1* or *GW182* dsRNA.

We have recently shown that oxygen-dependent subcellular localization is an important mechanism of Sima regulation: Sima shuttles continuously between the nucleus and cytoplasm, and nuclear export is inhibited in hypoxia [6], [40]. To get insights into how Ago1 depletion affects the transcriptional response to hypoxia, we studied Sima subcellular localization, and found no differences between Ago1 mutant embryos and wild type controls (Figure S3). Next we sought to study if Sima protein accumulation in hypoxic cells is affected upon Ago1 depletion. As depicted in Figure 3A, hypoxic induction of Sima protein is clearly reduced in S2 cells treated with *ago1* dsRNA. The next step was to analyze *sima* transcript levels. Real time PCR analysis revealed a striking upregulation of *sima* mRNA levels upon exposure of the cells to hypoxia, and that *ago1* RNAi treatment inhibited this induction (Figure 3B). These results indicate that HIF transcriptional induction or mRNA stabilization plays a role in the *Drosophila* hypoxic response, and suggests that the miRNA machinery participates in this regulation.

**Figure 4:**
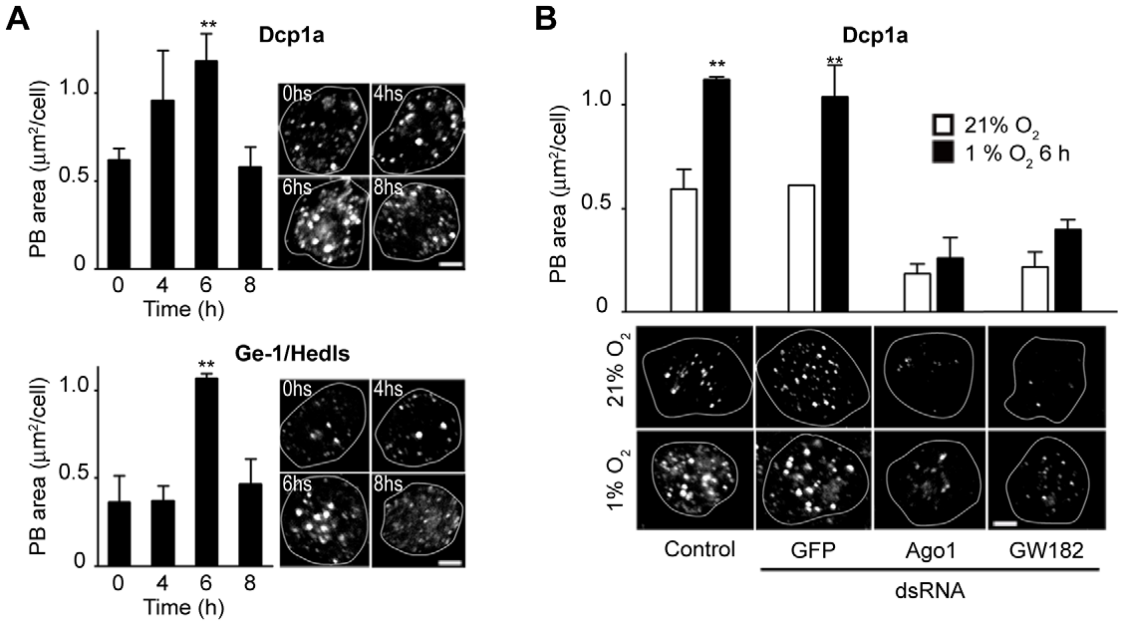
PBs accumulate in cells exposed to hypoxia in an Ago1- and GW182-dependent manner. (A) S2R+ cells were maintained in normoxia or exposed to hypoxia (1% O_2_) for different time periods, then fixed and stained with an anti-DCP1 or anti-Hedls antibodies, two PBs specific markers. The PB area per cell was determined, revealing that PBs accumulate in a transient manner in cells exposed to hypoxia, peaking at 6 h after the onset of the hypoxic treatment, and decreasing at 8 h (one-way ANOVA and Dunnett multiple comparison post-Test, **p<0.01). (B) *ago1* or *GW182* dsRNA treatment affect PB basal levels and prevent PB accumulation upon exposure of the cells to 1% O_2_ for 6 h. (one-way Anova and SNK multiple comparisons post-test, p<0.01).

The above results prompted us to analyze possible changes in the miRNA machinery in hypoxia: a well known effect of miRNA dependent translational silencing is the accumulation of Processing Bodies (PBs), which are cytosolic *foci* that contain untranslated mRNAs and proteins, as well as small RNAs involved in translational silencing [41]. Thus, we explored whether exposure to hypoxia stimulates accumulation of PBs. As shown in Figure 4A and 4B, a clear increase of PBs was observed after exposing the cells to hypoxia, as revealed by anti-DCP1 or anti-Hedls antibody staining [42]. The accumulation of PBs in hypoxia was transient, reaching a peak 6 hours after transferring the cells to 1% O_2_ (Figure 4A). To explore if this effect is related to the miRNA pathway, we analyzed PB formation in cells depleted of Ago1 or GW182 and exposed to hypoxia. As shown in Figure 4B, both basal PB levels and induction of PBs by hypoxia were strongly reduced in these cells. It is unclear whether PB accumulation *per se* is required for HIF-dependent transcription or if alternatively, PB accumulation only reflects the activity of the miRNA machinery in the hypoxic response.

**Figure 5:**
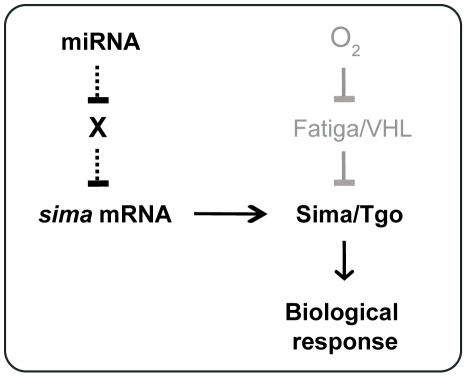
Model for *sima* regulation by the miRNA machinery. An unknown (“X”) factor that directly or indirectly inhibits *sima* transcription is silenced by the miRNA machinery. When cells are depleted from Ago1, the factor X accumulates thereby preventing *sima* transcriptional induction in hypoxia.

Taken together, our results suggest that the miRNA pathway plays a physiological role in cellular responses to hypoxia. Why does Ago1 depletion prevent *sima* mRNA induction? Although the identity of the target molecules that are controlled by the miRNA machinery is unknown, we can speculate that such unknown regulators directly or indirectly prevent *sima* transcriptional induction or alternatively contribute to *sima* messenger degradation (Figure 5). In mammalian cells, HIFα mRNAs are induced by NF-κB, so that NF-κB regulation plays an important role in the response to hypoxia [43]. It is not known if in *Drosophila* an NF-κB protein is required for *sima* transcriptional induction. If this was the case, it should be investigated if an inhibitor of the NF-κB pathway (i.e. IκB/Cactus) is subjected to miRNA-dependent translational regulation during adaptation to hypoxia.

Most major HIF regulators including PHDs, the von Hippel-Lindau tumour suppressor protein (VHL) [2] and factor inhibiting HIF (FIH) [4] are all inhibitors of the hypoxic response. The screen we have carried out here was instead focused on positive regulators of HIF. One remarkable feature of the results we have obtained is that most of the hits belong to just a few multiprotein complexes or signaling pathways. These include the PI3K/TOR pathway (translational regulation), eIF3 and eEF2 complexes (translational regulation), the COP9 signalosome (protein degradation/translational regulation), and the Brahma/SWI/SNF complex (chromatin remodeling). Noteworthy, besides genes belonging to these complexes, other hits of the screen are also linked to translational control (Ago1) or chromatin remodeling (Reptin, Pontin). Thus, one central conclusion of the results of this screen is that translational control and chromatin remodeling are two important mechanisms of HIF regulation, whose characterization in detail will broaden our understanding of HIF regulation and the cellular response to hypoxia.

## Materials and Methods

### Vectors, reporters, and cell culture

The reporter plasmids HRE- *firefly* luciferase (HRE-Luc) and act-*renilla* luciferase were previously described [15], [44]. The miRNA reporter pAC-miR-12 and CG10011-luc were a gift from E. Izaurralde [45]. pBLAST (Blasticidine resistance) and pPUR (Puromycin resistance) vectors were used to generate S2 stable cell lines. *Drosophila* Schneider's lines S2 or S2R^+^ cells were maintained at 25°C in Schneider or M3 medium (Sigma), supplemented with 10% fetal bovine serum (Gibco), 50 units/ml penicillin and 50 µg/ml streptomycin in 25 or 75 cm^2^ T-flasks (Greiner). Cells were grown in 12, 24, 96 or 384-well plates (Greiner), during 3 days and treated with 100 µM of DFO (Sigma) or exposed to hypoxia in a Forma Scientific 3131 incubator.

### Synthesis of dsRNA and RNAi treatments

For dsRNAs not obtained from the *Drosophila RNAi Screening Center* (DRSC), fragments of the genes were amplified by PCR from cDNA or genomic DNA using T7-tailed oligonucleotides as primers. dsRNA synthesis was carried out with a T7 Megascript kit (Ambion) following manufacturer's instructions. The “bathing” method was utilized to introduce dsRNAs into S2 or S2R^+^ cells as previously described [46].

### RNAi Screens

For screening experiments *Drosophila* S2 cells were maintained at 25°C in Schneider's medium. The primary screen was carried out at the *Drosophila RNAi Screening Center* (DRSC), the secondary and tertiary screens were performed in our laboratory with dsRNAs obtained from the DRSC. Primer and amplicon information can be found at http://flyrnai.org.

#### Primary screen

Two sets of 58 384-well screening plates (Costar) containing approximately 0.2 µg of dsRNA per well were provided by the DRSC (DRSC 1.0 library). Sima or GFP were used as positive and negative controls, respectively. Three days after plating, the cells were stimulated with DFO (100 µM) for 20 h and then *firefly luciferase* activity was determined using the SteadyGlo reagent (Promega). The Z-score value for each well was calculated as the luciferase activity of the well minus the average of the luciferase activity of the whole plate divided by the standard deviation of the plate.

#### Secondary screen

The secondary screen was carried out in 96-well plates. *Firefly* and *Renilla* luciferase activities were determined using the DualGlo reagent (Promega) in a Veritas Luminometer. Normalized luciferase activity (*firefly* to *renilla* luciferase activity ratio) for each well was calculated as a percentage of the control wells treated with GFP dsRNA.

#### Tertiary screen in hypoxia

Fifty-nine dsRNAs from the DRSC Validation library were used to cover the 35 selected genes. S2-HRE-Luc cells were incubated with dsRNAs in 96-well plates as described above and then exposed to hypoxia (1% O_2_) in a Forma Scientific 3131 incubator for 20 hours. *Firefly* and *Renilla* luciferase activities were determined and normalized as above.

### Real-time PCR

Total RNAs from cells exposed to different treatments were isolated using the Trizol reagent (Invitrogen). One to 5 µg of total RNA were used as a template for cDNA synthesis, using the SuperScript III First Strand Synthesis System for RT-PCR (Invitrogen). Quantitative real time PCR was performed in the MX3005P real time PCR instrument (Stratagene, La Jolla, CA) with Syber Green, the hot start Platinum Taq DNA polymerase (Invitrogen) and the ROX reference dye (Invitrogen). Primers for amplifying 100–300 bp of each PCR product were used. PCR reactions were carried out for 5 min at 95°C followed by 35 cycles of three-step PCR for 30 seconds at 95°C, 1 min at 60°C, and 1 min at 72°C. Each sample was analyzed in triplicate. The data were normalized by subtracting the difference of the C_T_ values between the target gene of interest (Tgene) and that of tubulin mRNA, thereby obtaining a ΔC_T_ (Tgene C_T_ −Tubulin C_T_). Relative expression (fold induction) was calculated as 2^−(SΔC^_T_^−CΔC^_T_^)^ where SΔC_T_−CΔC_T_ is the difference between the sample ΔC_T_ (treated cells) and the control ΔC_T_ (RNAi GFP cells). Both target gene and tubulin reactions approached 100% efficiency as determined by standard curves. PCR products were analyzed on agarose gels to check that a single band was amplified.

### Fly stocks

Flies used were *yw*, *ldh*- LacZ [12] and *yw*, *ago1^k08121^*/*CyO*.

### β-galactosidase activity

Wild type or *ago1^k08121^* embryos were exposed to 3% or 21% O_2_ for 4 h, homogenized in lysis buffer (50 mM Tris HCl [pH 7.8], 2 mM EDTA, 10% glycerol, 2 mM DTT, 1% Triton X-100, 1 mM PMSF) and centrifuged at 2,500×g for 3 min at 4°C. Enzymatic reactions were carried out by incubating 20 to 50 µg of protein extract in 180 µl buffer, containing 80 mM Na3PO4 (pH 7.3), 102 mM β-mercaptoethanol, 9 mM MgCl2, and 4 mM CPRG (Chlorophenol Red β-d-galactopyranoside; Roche Diagnostics) at 37°C, and absorbance at 574 nm was recorded at 10, 30, 60, 120, and 180 min time points; color development was linear throughout this time period. Endogenous background was subtracted using a heat-inactivated sample.

### Immunofluorescence

For PB staining either a mouse monoclonal anti-DCP1 antibody (Abnova) was utilized at a 1∶1000 dilution were used or a rabbit anti-HEDLS antiserum (Bethyl) was used at 1∶500 dilution. Images were acquired in LSM510 Meta confocal microscopes (Carl Zeiss), using a Plan-Apochromat 63×/1.4 oil objective. Equipment adjustment was assessed by using 1µm Focal Check fluorescent microspheres (Molecular Probes). Pictures were exported to Adobe Photoshop software for cropping. Neither filters nor gamma-adjustments were applied. PB number and size in µm^2^ were determined with the “Analyze Particles” tool of the Image J software (NIH) in randomly selected micrographs.

## Supporting Information

Figure S1 HRE-luciferase reporter induction in cells exposed to hypoxia or DFO. (A) Schematic representation of the HIF-responsive firefly luciferase reporter element used in this study (HRE-Luc). A dimerized regulatory sequence derived from the murine lactate dehydrogenase enhancer was cloned upstream of a firefly luciferase gene in a pGL3 plasmid bearing a fly hsp70 minimal promoter. Each 51 bp sequence contains two HIF responsive elements (HREs) and one cyclic AMP responsive element (CRE). (B) S2-HRE-luc cells were seeded in 96-wells tissue culture plates (1×104 cells per well), grown for 3 days, and stimulated with DFO (100 µM), or exposed to hypoxia (1% O2) for 20 hours. Strong induction of luciferase activity was observed in cells stimulated with DFO or hypoxia. Results are expressed as fold induction of luciferase activity respect to normoxic untreated cells. (C) Scatter plot of the duplicate results (Z-scores; see Materials and Methods) of the primary screen, showing the overall reproducibility of the data.
(0.03 MB PDF)

Figure S2 miRNAs and the response to hypoxia. (A) Upper panel, schematic representation of the miRNA reporter CG10011-luc; the *miR-12* miRNA binds to the 3′ UTR of the *luciferase* mRNA, thereby inhibiting translation. Over-expression of miR-12 is therefore expected to provoke strong inhibition of translation. Lower panel, S2 cells were co-transfected with the CG10011-luc reporter and the pAC-miR-12 over-expression plasmid, or with an empty vector (pAC) as a control, and exposed to *ago1* or *gfp* dsRNA treatments during 4 days. miR-12 over-expression inhibits 80% of luciferase expression in the control cells treated with *gfp* dsRNA, whereas in cells depleted from *ago1* (*ago1.1* or *ago1.2* dsRNAs) miR-12 over-expression failed to inhibit luciferase expression to a large extent. (B) S2-HRE-luc cells were treated with dsRNA against *gfp* (control), *sima*, *ago1*, *ago2*, *piwi*, or *dicer-2*, grown during 4-8 days, and stimulated with DFO (100 µM). Cells depleted from *ago1* or *sima* showed strong reduction of reporter activity, whereas cells depleted from *ago2*, *dicer-2*, or *piwi* exhibited normal induction of the reporter upon DFO exposure.
(0.01 MB PDF)

Figure S3 Regulation of Sima subcellular localization is not affected in Ago1 homozygous mutant embryos. We have analyzed Sima subcellular localization in en-Gal4/UAS-sima transgenic embryos carrying a homozygous mutation in the Ago1 locus (*ago1^k0208^*), and compared with Sima localization in en-Gal4/UAS-sima wild type individuals. The analysis was carried out as we reported previously (Dekanty et al., 2005) [15]. Three categories of Sima subcellular localization were defined for quantitative purposes: “Nuclear” (black color), “Ubiquitous” (grey) and “Cytoplasmic” (white). The Ago1 mutation does not impinge on Sima subcellular localization neither in normoxia nor in hypoxia.
(0.01 MB PDF)

Table S1 Summary of the three phases of the overall screen for genes required for HIF activity.
(0.04 MB PDF)

Table S2 Results of the primary screen carried out in cells exposed to DFO are shown. The screen was performed in duplicate; genes in which at least one of the two Z scores values was below −2.5 are depicted in the table. Under this criterion, 603 genes scored as positives in this initial phase of the screen.
(0.43 MB PDF)

Table S3 The data obtained at the primary screen (Table S2) were filtered against the results of a cell viability screen previously carried out at the DRSC (Boutros et al. 2004) [23]. Sequences from the “Sanger collection” were also eliminated from the study; the 225 genes that remained as positive hits of the primary screen are depicted.
(0.27 MB PDF)

Table S4 The secondary screen was also carried out in cells exposed to DFO. A second-generation library (DRSC 2.0 library) was used, in which most genes are represented by more than one dsRNA. Normalized luciferase activity (*firefly*/*renilla* luciferase activity ratio) for each well was calculated and expressed as a percentage of the inhibition respect to control cells treated with dsRNA against GFP that were exposed to DFO. The screen was carried out in duplicate and the mean percentage of inhibition is depicted.
(0.26 MB PDF)
