# Supplementary figures and images for: Drosophila Genome-Wide RNAi Screen Identifies Multiple Regulators of HIF–Dependent Transcription in Hypoxia
Source: PLoS Genet. 2010 Jun 24;6(6):e1000994. doi: 10.1371/journal.pgen.1000994 (PMC2891703; doi:10.1371/journal.pgen.1000994)

Figure S1. HRE-Luciferase reporter induction in cells exposed to hypoxia or DFO.

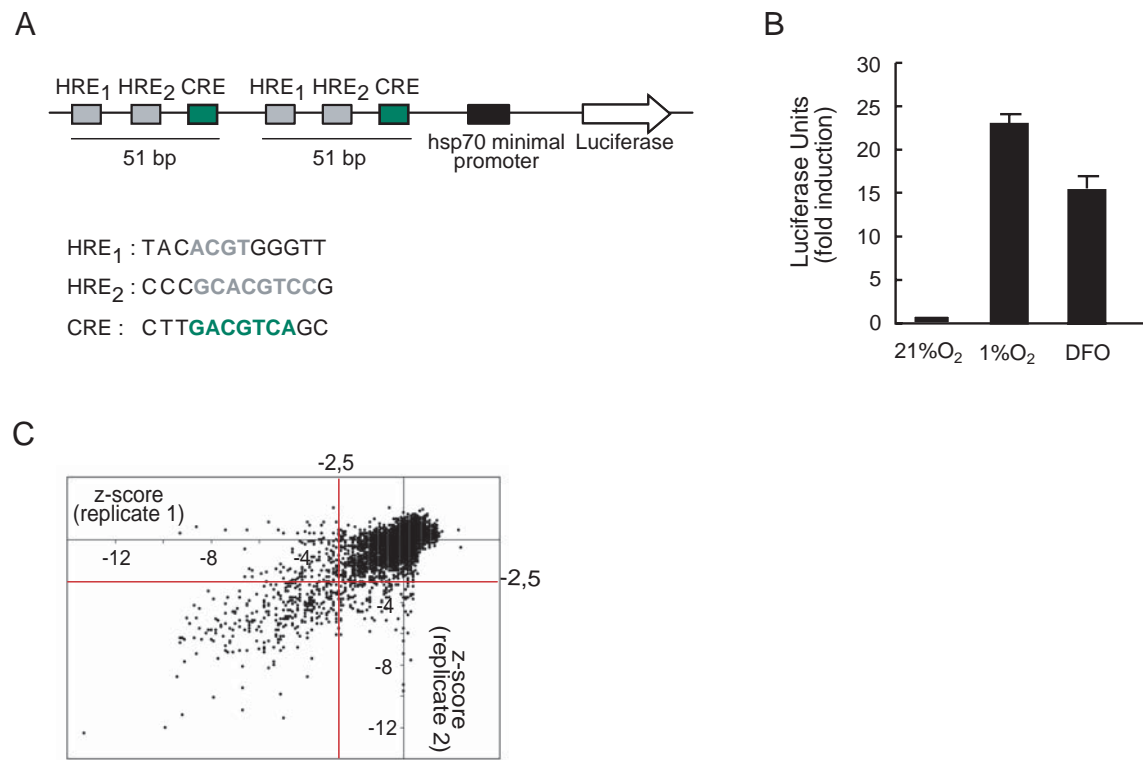

Supplement: Figure S1 — HRE-luciferase reporter induction in cells exposed to hypoxia or DFO. (A) Schematic representation of the HIF-responsive firefly luciferase reporter element used in this study (HRE-Luc). A dimerized regulatory sequence derived from the murine lactate dehydrogenase enhancer was cloned upstream of a firefly luciferase gene in a pGL3 plasmid bearing a fly hsp70 minimal promoter. Each 51 bp sequence contains two HIF responsive elements (HREs) and one cyclic AMP responsive element (CRE). (B) S2-HRE-luc cells were seeded in 96-wells tissue culture plates (1×10^4^ cells per well), grown for 3 days, and stimulated with DFO (100 µM), or exposed to hypoxia (1% O2) for 20 hours. Strong induction of luciferase activity was observed in cells stimulated with DFO or hypoxia. Results are expressed as fold induction of luciferase activity respect to normoxic untreated cells. (C) Scatter plot of the duplicate results (Z-scores; see Materials and Methods) of the primary screen, showing the overall reproducibility of the data. (0.03 MB PDF) [file pgen.1000994.s001.pdf]

**Figure S2. miRNAs and the response to hypoxia.**

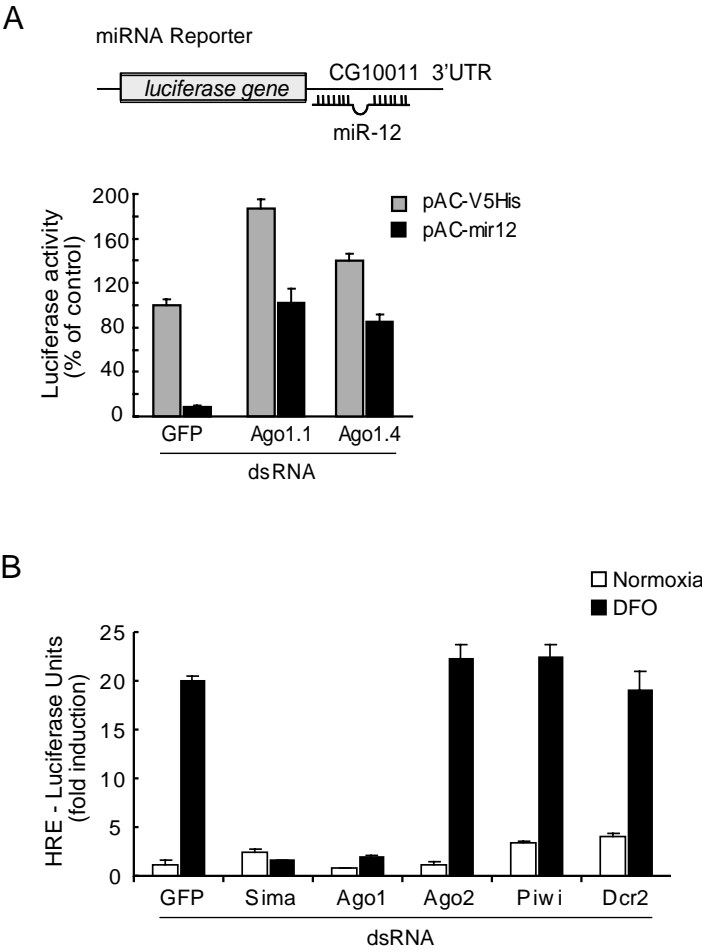

Supplement: Figure S2 — miRNAs and the response to hypoxia. (A) Upper panel, schematic representation of the miRNA reporter CG10011-luc; the miR-12 miRNA binds to the 3′ UTR of the luciferase mRNA, thereby inhibiting translation. Over-expression of miR-12 is therefore expected to provoke strong inhibition of translation. Lower panel, S2 cells were co-transfected with the CG10011-luc reporter and the pAC-miR-12 over-expression plasmid, or with an empty vector (pAC) as a control, and exposed to ago1 or gfp dsRNA treatments during 4 days. miR-12 over-expression inhibits 80% of luciferase expression in the control cells treated with gfp dsRNA, whereas in cells depleted from ago1 (ago1.1 or ago1.2 dsRNAs) miR-12 over-expression failed to inhibit luciferase expression to a large extent. (B) S2-HRE-luc cells were treated with dsRNA against gfp (control), sima, ago1, ago2, piwi, or dicer-2, grown during 4-8 days, and stimulated with DFO (100 µM). Cells depleted from ago1 or sima showed strong reduction of reporter activity, whereas cells depleted from ago2, dicer-2, or piwi exhibited normal induction of the reporter upon DFO exposure. (0.01 MB PDF) [file pgen.1000994.s002.pdf]
