# Supplementary material for: Drosophila Genome-Wide RNAi Screen Identifies Multiple Regulators of HIF–Dependent Transcription in Hypoxia
Source: PLoS Genet. 2010 Jun 24;6(6):e1000994. doi: 10.1371/journal.pgen.1000994 (PMC2891703; doi:10.1371/journal.pgen.1000994)

Figure S3. Regulation of Sima subcellular localization is not affected in Ago1 homozygous mutant embryos.

A

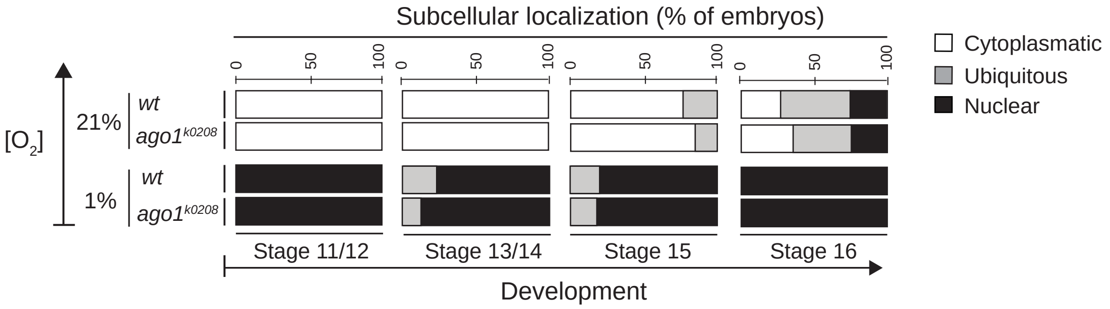

Supplement: Figure S3 — Regulation of Sima subcellular localization is not affected in Ago1 homozygous mutant embryos. We have analyzed Sima subcellular localization in en-Gal4/UAS-sima transgenic embryos carrying a homozygous mutation in the Ago1 locus (ago1k0208), and compared with Sima localization in en-Gal4/UAS-sima wild type individuals. The analysis was carried out as we reported previously (Dekanty et al., 2005) [15]. Three categories of Sima subcellular localization were defined for quantitative purposes: “Nuclear” (black color), “Ubiquitous” (grey) and “Cytoplasmic” (white). The Ago1 mutation does not impinge on Sima subcellular localization neither in normoxia nor in hypoxia. (0.01 MB PDF) [file pgen.1000994.s003.pdf]
