# Supplementary material for: Drosophila Genome-Wide RNAi Screen Identifies Multiple Regulators of HIF–Dependent Transcription in Hypoxia
Source: PLoS Genet. 2010 Jun 24;6(6):e1000994. doi: 10.1371/journal.pgen.1000994 (PMC2891703; doi:10.1371/journal.pgen.1000994)
Supplement: Table S1 — Summary of the three phases of the overall screen for genes required for HIF activity. (0.04 MB PDF) [file pgen.1000994.s004.pdf]

Table S1. Summary of the three phases of the overall screen for genes required for HIF activity.

Screen	Genes Analyzed	Reporter System	dsRNA library	Stimulus	Hit Selection Criterion	N° of hits
**Primary**	~22,000 genes (Whole DRSC 1.0 dsRNA library)	HRE- firefly Luc	DRSC 1.0	DFO	Z score ≤ -2,5	609
**Secondary**	225 Primary hits (Total primary hits filtered against cell viability screen and Sanger Collection)	HRE- firefly Luc and Actin- *Renilla* Luc	DRSC 2.0	DFO	Inhibition of HRE-luc activity ≥ 50% (Group A >75% ; Group B 50%-75%)	66
**Tertiary**	35 Secondary hits (Group A and 12 genes from Group B functionally related to Group A genes)	HRE- firefly Luc and Actin- *Renilla* Luc	DRSC 2.0	Hypoxia (1% O_2)	Inhibition of HRE-luc activity ≥ 50%	30
