# Supplementary material for: Drosophila Genome-Wide RNAi Screen Identifies Multiple Regulators of HIF–Dependent Transcription in Hypoxia
Source: PLoS Genet. 2010 Jun 24;6(6):e1000994. doi: 10.1371/journal.pgen.1000994 (PMC2891703; doi:10.1371/journal.pgen.1000994)
Supplement: Table S2 — Results of the primary screen carried out in cells exposed to DFO are shown. The screen was performed in duplicate; genes in which at least one of the two Z scores values was below −2.5 are depicted in the table. Under this criterion, 603 genes scored as positives in this initial phase of the screen. (0.43 MB PDF) [file pgen.1000994.s005.pdf]

Table S2. Primary screen.

Primary screen				
Gene	Amplicon	-Z1	-Z2	Average - Z
HDC05561 ('+' in Hild et al)	DRSC05209	13.3	12.4	12.9
RpL15	DRSC20963	12.7	9.7	11.2
HDC06418 ('+' in Hild et al)	DRSC05498	9.2	11.2	10.2
no hitter	DRSC01976	9.3		9.3
HDC17524 ('+' in Hild et al)	DRSC17394	9.4	8.8	9.1
RpL19	DRSC04649	7.9	10.1	9.0
HDC07108 ('+' in Stolc / Hild et al)	DRSC05715	8.5	9.3	8.9
HDC07858 ('+' in Hild et al)	DRSC07932	6.7	10.9	8.8
Pdp1	DRSC08897	8.6		8.6
brahma	DRSC11330	7.8	9.1	8.5
RpS26	DRSC03420	6.0	10.5	8.3
HDC14318 ('+' in Hild et al)	DRSC13298	11.4	5	8.2
Pomp	DRSC03201	6.7	9.5	8.1
RpL21	DRSC03704	6.6	9.3	8.0
CG10660	DRSC09769	7.3	8.6	8.0
cyclophilin-33	DRSC07600	6.3	9.3	7.8
hoi-polloi	DRSC03546	6.4	9.1	7.8
RpL12	DRSC04344	6.3	9.2	7.8
CG4174	DRSC10406	7.7		7.7
HDC18812 ('+' in Hild et al)	DRSC18999	7.7	7.6	7.7
HDC15690 ('+' in Hild et al)	DRSC13740	8.7	6.3	7.5
RpL18	DRSC11016	5.1	9.9	7.5
RpS12	DRSC11270	6.4	8.4	7.4
MED11	DRSC10739	8.1	6.7	7.4
CG4587	DRSC01956	7.4		7.4
RpL26	DRSC10726	7.2	7.3	7.3
RpL35A	DRSC12302	7.1	7.4	7.3
RpL7	DRSC03417	6.4	8	7.2
RpL23	DRSC04648	6.5	7.9	7.2

CG2807	DRSC00535	7.2	7.1	7.2
RpS19a	DRSC20281	5.4	8.9	7.2
Tbp-1	DRSC16842	5.7	8.5	7.1
RpL8	DRSC08695	6.8	7.4	7.1
RpL14	DRSC11269	6.0	8.2	7.1
HDC12613 ('+' in Hild et al)	DRSC12723	5.0	9	7.0
Pros26.4	DRSC16799	5.8	8.1	7.0
HDC17815 ('+' in Hild et al)	DRSC17506	6.8	7.1	7.0
eIF-4G	DRSC17218	6.0	7.8	6.9
Rpn11	DRSC03422	6.6	7.2	6.9
CG11985	DRSC14460	5.8	8	6.9
RpL32	DRSC16835	5.6	8.2	6.9
CG9119	DRSC08620	6.5	7.2	6.9
RpL8	DRSC08532	6.2	7.5	6.9
RpS9	DRSC11273	5.8	7.8	6.8
eIF-4a	DRSC03526	5.0	8.6	6.8
RpL10Ab	DRSC10798	7.1	6.5	6.8
RpL22	DRSC18707	5.4	8	6.7
quemao	DRSC11947	6.2	7.1	6.7
RpS13	DRSC03419	5.4	7.8	6.6
RpL38	DRSC07818	5.6	7.6	6.6
Rpn7	DRSC16841	5.5	7.6	6.6
RpL39	DRSC04651	7.0	6	6.5
HDC12958	DRSC12832	7.2	5.8	6.5
CG15278	DRSC02481	6.5		6.5
RpS18	DRSC07540	5.1	7.8	6.5
RpS4	DRSC11272	5.2	7.7	6.5
RpS14b	DRSC18711	4.4	8.5	6.5
RpS15Ab, RpS15Aa	DRSC19831	4.2	8.7	6.5
Dsp1	DRSC20315	5.8	7.1	6.5
CG32000	DRSC17103	5.7	7.2	6.5
RpL5	DRSC03801	5.4	7.4	6.4
eIF3-S10	DRSC12339	5.4	7.4	6.4
sima	DRSC17055	7.0	5.8	6.4
RpL13	DRSC03416	4.0	8.8	6.4
RpL27	DRSC15638	6.9	5.8	6.4
CG9134	DRSC07954	6.3		6.3
Trip1	DRSC03464	6.1	6.5	6.3
RpL27A	DRSC00781	6.5	6.1	6.3
CG3605	DRSC00619	5.8	6.6	6.2
RpL4	DRSC16833	5.8	6.6	6.2
CG18591	DRSC02680	5.6	6.8	6.2
RpL3	DRSC16834	5.4	7	6.2

RpL18A	DRSC07538	5.9	6.4	6.2
HDC18154 ('+' in Hild et al)	DRSC17612	5.4	6.9	6.2
RpL24	DRSC03185	5.1	7.1	6.1
combgap	DRSC07650	7.1	5.1	6.1
HDC07436 ('+' in Hild et al)	DRSC05853	5.7	6.5	6.1
Mov34	DRSC04624	6.5	5.6	6.1
pixie	DRSC10533	5.7	6.4	6.1
Pros25	DRSC16798	4.7	7.4	6.1
sop	DRSC03614	6.8	5.2	6.0
arrest	DRSC01234	4.7	7.3	6.0
CG8636	DRSC18427	5.5	6.4	6.0
Rpn2	DRSC16839	5.8	6.1	6.0
RpS7	DRSC15394	6.9	5	6.0
Hsc70-4	DRSC16711	5.9	5.9	5.9
RpL32	DRSC16835	6.6	5.2	5.9
nucampholin	DRSC02179	7.9	3.8	5.9
RpS5a	DRSC20282	4.7	7	5.9
Ef2b	DRSC03737	5.2	6.5	5.9
Rpn12	DRSC11275	4.6	7.1	5.9
eIF3-p48/Int6	DRSC11199	5.3	6.4	5.9
Pros26	DRSC11256	5.3	6.3	5.8
RpL17	DRSC18293	4.6	7	5.8
eIF3-S8	DRSC06905	6.4	5.2	5.8
RpS17	DRSC11271	5.6	6	5.8
Rpt1	DRSC07542	6.4	5.1	5.8
RpS6	DRSC18712	4.1	7.4	5.8
RpS29	DRSC16433	5.2	6.3	5.8
RpS3	DRSC16838	6.2	5.3	5.8
Pros35	DRSC03401	6.1	5.3	5.7
Prosbeta5	DRSC07517	5.2	6.2	5.7
CG6694	DRSC10696	4.9	6.5	5.7
RpS30	DRSC15119	7.6	3.8	5.7
BarH2	DRSC19335	6.1	5.3	5.7
nAcRalpha-96Aa	DRSC13670	8.1	3.3	5.7
CG15494	DRSC02502	5.7		5.7
RpS15	DRSC07151	4.4	6.9	5.7
RpLP2	DRSC07539	6.2	5.1	5.7
RpS3A	DRSC17168	6.9	4.4	5.7
SmB	DRSC03437	5.9	5.3	5.6
RpS8	DRSC16318	6.2	5	5.6
Prosalpha7	DRSC07516	5.8	5.3	5.6
eIF-4E	DRSC11342	3.7	7.3	5.5
CG30089	DRSC06127	4.7	6.3	5.5

RpL36	DRSC18708	5.8	5.2	5.5
Rpn1	DRSC11274	5.0	5.8	5.4
RpL32	DRSC16835	5.2	5.6	5.4
Prosbeta3	DRSC16801	4.6	6.1	5.4
CG1161	DRSC12180	5.5	5.2	5.4
RpL7A	DRSC18709	4.2	6.5	5.4
Ataxin-2	DRSC15727	5.4	5.2	5.3
CG13235	DRSC06355	5.6	5	5.3
Rpt3	DRSC20283	4.1	6.5	5.3
CG9769	DRSC12328	5.4	5.1	5.3
moira	DRSC15378	5.2	5.3	5.3
HDC17859 ('+' in Hild et al)	DRSC17532	6.8	3.7	5.3
CG42400	DRSC02458	4.3	6.2	5.3
p130CAS	DRSC08257	5.5	4.9	5.2
NSL1	DRSC15625	4.8	5.6	5.2
seven up	DRSC15311	6.0	4.4	5.2
Dox-A2	DRSC03318	4.6	5.7	5.2
RpL35	DRSC18347	3.9	6.4	5.2
Helicase at 25E	DRSC03342	5.1	5.1	5.1
RpL9	DRSC03418	5.7	4.5	5.1
RpS14a	DRSC18710	4.6	5.6	5.1
CG15097	DRSC06526	5.1	5	5.1
Prosbeta2	DRSC11257	4.3	5.8	5.1
RpS15Ab, RpS15Aa	DRSC06129	3.3	6.8	5.1
RpL31	DRSC06716	4.8	5.3	5.1
RpII18	DRSC12366	5.5	4.6	5.1
eIF3-S9	DRSC06900	5.1	4.9	5.0
ran	DRSC20364	4.2	5.8	5.0
HDC09524 ('+' in Hild et al)	DRSC09077	4.4	5.6	5.0
HDC17206 ('+' in Stolc / Hild et al)	DRSC17248	6.0	4	5.0
oho23B	DRSC00833	4.6	5.4	5.0
RpL40	DRSC00782	5.0	4.9	5.0
eIF-3p66	DRSC16938	5.1	4.8	5.0
DebB	DRSC07397	5.0	4.8	4.9
Rrp6	DRSC16223	4.2	5.6	4.9
Cyclin E	DRSC03296	4.4	5.4	4.9
Rpb5	DRSC06100	4.0	5.8	4.9
Rpt4	DRSC18713	4.9	4.9	4.9
CG5931	DRSC10559	4.1	5.6	4.9
thread	DRSC11404	4.9	4.8	4.9
SmD3	DRSC07553	3.9	5.8	4.9
dos	DRSC07992	5.3	4.4	4.9
abdominal A	DRSC16897	5.0	4.6	4.8

RpS20	DRSC16836	5.8	3.8	4.8
HDC02682 ('+' in Hild et al)	DRSC01481	6.1	3.5	4.8
squid	DRSC17066	4.8		4.8
CG13550	DRSC04191	4.5	5	4.8
CG14459	DRSC11719	5.3	4.2	4.8
raptor	DRSC18359	4.7		4.7
double parked	DRSC07088	4.9	4.5	4.7
CG42342	DRSC14378	4.2	5.2	4.7
prp8	DRSC07293	5.3	4.1	4.7
CG7065	DRSC18420	3.6	5.7	4.7
eIF-5A	DRSC04681	4.5	4.8	4.7
RpL28	DRSC08293	4.8	4.5	4.7
RpS28b	DRSC18258	4.6	4.6	4.6
CG12912	DRSC06227	4.5	4.7	4.6
HDC16553 ('+' in Hild et al)	DRSC14046	4.6	4.6	4.6
CG16941	DRSC15166	3.7	5.4	4.6
murashka	DRSC16484	4.2	4.9	4.6
Tango7	DRSC07142	5.2	3.8	4.5
Rpn6	DRSC07541	4.0	5	4.5
lethal(2)05070	DRSC07159	5.4	3.6	4.5
CG17331	DRSC02603	4.6	4.4	4.5
CG17836	DRSC15282	4.9	4.1	4.5
HDC09397 ('+' in Stolc / Hild et al)	DRSC09009	6.6	2.4	4.5
CG5482	DRSC06936	6.1	2.8	4.5
CG8929	DRSC07298	3.8	5.1	4.5
Prosbeta4	DRSC12186	4.5	4.4	4.5
Ken and Barbie	DRSC04696	5.8	3	4.4
CG4849	DRSC15662	5.0	3.8	4.4
CG17304	DRSC15222	4.7	4.1	4.4
CG15450	DRSC20522	4.6	4.2	4.4
Spt6	DRSC18836	4.0	4.8	4.4
HDC07396 ('+' in Hild et al)	DRSC05818	3.4	5.4	4.4
AGO1	DRSC05912	6.1	2.6	4.4
RpII215	DRSC20280	3.5	5.2	4.4
chinmo	DRSC00509	3.6	5.1	4.4
Nup98	DRSC14210	4.1	4.6	4.4
CG30382	DRSC07515	4.2	4.4	4.3
RpS16	DRSC04442	4.5	4.1	4.3
CG12897	DRSC06212	3.4	5.2	4.3
SF2	DRSC16845	3.2	5.4	4.3
HDC01261 ('+' in Stolc / Hild et al)	DRSC00977	2.9	5.7	4.3
HDC02560 ('+' in Hild et al)	DRSC01970	3.6	5	4.3
CSN6	DRSC16593	4.3		4.3

Jon99Ciii	DRSC16859	4.3		4.3
AdoR	DRSC16556	4.3		4.3
crooked-neck	DRSC18755	3.9	4.7	4.3
cdc2	DRSC03504	4.0	4.5	4.3
Patj	DRSC08712	4.0	4.5	4.3
Suv4-20	DRSC18482	3.9	4.6	4.3
snRNP69D	DRSC09800	5.7	2.7	4.2
TER94	DRSC07560	4.5	3.9	4.2
Bx42	DRSC17743	4.9	3.5	4.2
Nup153	DRSC19904	2.9	5.5	4.2
CG32365	DRSC10041	3.7	4.7	4.2
CG4328	DRSC10410	4.6	3.8	4.2
pontin	DRSC17029	4.5	3.9	4.2
ascutex	DRSC05924	2.7	5.7	4.2
HDC19639 ('+' in Stolc / Hild et al)	DRSC19272	3.1	5.3	4.2
orb	DRSC17021	4.5	3.8	4.2
Nup153	DRSC19904	5.5	2.8	4.2
RpL30	DRSC02087	3.7	4.6	4.2
reptin	DRSC11388	5.1	3.2	4.2
small bristles	DRSC20368	4.2	4.1	4.2
CG7757	DRSC10912	5.1	3.2	4.2
CG33967	DRSC16257	4.7	3.6	4.2
HDC13910	DRSC13145	4.1	4.2	4.2
HDC15172 ('+' in Hild et al)	DRSC13607	6.3	2.0	4.2
CG32606	DRSC19001	6.4	1.9	4.2
tango	DRSC17077	3.3	4.8	4.1
Bap60	DRSC19337	3.8	4.4	4.1
Slu7	DRSC14729	3.7	4.5	4.1
CG4266	DRSC04456	4.1	4.1	4.1
Tango4	DRSC19786	3.9	4.3	4.1
CG13298	DRSC09972	3.3	4.9	4.1
CG7918	DRSC16345	5.9	2.2	4.1
HDC03687 ('+' in Hild et al)	DRSC03744	5.3	2.9	4.1
Trn-SR	DRSC00546	3.7	4.4	4.1
CG17742	DRSC10285	5.3	2.8	4.1
Su(var)3-9	DRSC13081	4.0	4.1	4.1
eIF-1A	DRSC16937	4.7	3.4	4.1
His3:CG31613	DRSC21267	5.9	2.2	4.1
starry night	DRSC05234	4.1	4	4.1
ph-d	DRSC18819	4.0		4.0
CG14641	DRSC12227	3.1	4.9	4.0
RpLP1	DRSC00783	4.8	3.2	4.0
CG13165	DRSC06292	4.2	3.8	4.0

CG13675	DRSC10034	4.1	3.9	4.0
RpII15	DRSC16832	3.7	4.3	4.0
CG4325	DRSC18516	4.3	3.7	4.0
CG14180	DRSC10179	4.0	3.9	4.0
CG34139	DRSC15701	4.0		4.0
CG6197	DRSC06967	4.1	3.8	4.0
CG1542	DRSC15035	4.8	3.2	4.0
Nup153	DRSC19904	2.8	5.1	4.0
Pvf2	DRSC00968	2.6	5.2	3.9
Calx	DRSC13457	3.9		3.9
Neosin	DRSC11235	3.5	4.3	3.9
RpL36A	DRSC03055	4.3	3.5	3.9
Ef1alpha48D	DRSC07421	3.5	4.3	3.9
CG14952	DRSC08412	3.9	3.9	3.9
HDC03105 ('+' in Hild et al)	DRSC01651	3.9		3.9
ind	DRSC11355	3.0	4.8	3.9
Rheb	DRSC12148	3.0	4.7	3.9
CG14995	DRSC08452	4.6	3.1	3.9
MED25	DRSC14483	4.4	3.3	3.9
HDC05695	DRSC06151	3.9	3.8	3.9
Cpr47Ee	DRSC06343	5.1	2.6	3.9
unc-13-4A	DRSC10203	4.0	3.7	3.9
stubarista	DRSC18838	3.8	3.9	3.9
CG30126	DRSC05769	3.6	4.1	3.9
HDC11530	DRSC11566	4.9	2.8	3.9
smt3	DRSC03611	4.5	3.1	3.8
Fs(2)Ket	DRSC03328	3.9	3.7	3.8
gawky	DRSC17160	4.8	2.8	3.8
CR11700	DRSC17794	3.9	3.7	3.8
CG8179	DRSC07091	4.8	2.8	3.8
CG12499	DRSC14521	3.8		3.8
Chrac-16	DRSC20343	3.8		3.8
U2af50	DRSC20297	3.5	4	3.8
HDC05705 ('+' in Stolc / Hild et al)	DRSC06353	3.7	3.8	3.8
diminutive	DRSC18762	4.7	2.8	3.8
HDC10026	DRSC09271	2.8	4.7	3.8
HDC14006	DRSC13177	4.3	3.2	3.8
Rdh	DRSC08434	5.0	2.5	3.8
HDC12966 ('+' in Hild et al)	DRSC14922	3.8	3.7	3.8
Pvr	DRSC03080	2.9	4.5	3.7
bruno-2	DRSC02557	2.8	4.6	3.7
bric a brac 1	DRSC08379	3.0	4.4	3.7
minibrain	DRSC20058	2.7	4.7	3.7

blistered	DRSC04676	4.4	3	3.7
HDC02560 ('+' in Hild et al)	DRSC01970	3.7	3.6	3.7
Pros29	DRSC04644	4.0	3.3	3.7
RpS24	DRSC04414	5.9	1.4	3.7
Ef1gamma	DRSC16659	2.8	4.5	3.7
l(3)03670	DRSC16981	3.6		3.6
stumps	DRSC15332	5.3	1.9	3.6
Rtf1	DRSC04085	3.8	3.4	3.6
HDC05705 ('+' in Stolc / Hild et al)	DRSC06353	3.5	3.7	3.6
split thorax	DRSC18720	2.6	4.6	3.6
HDC07387 ('+' in Hild et al)	DRSC05810	4.3	2.9	3.6
HDC09490 ('+' in Hild et al)	DRSC09057	5.1	2.1	3.6
CG31395	DRSC12788	6.6	0.6	3.6
RpS10b	DRSC19561	5.1	2.1	3.6
CG32772	DRSC18052	4.6	2.6	3.6
Cyclin T	DRSC11124	4.2	2.9	3.6
His4r	DRSC16703	3.3	3.8	3.6
Tcp1-like	DRSC16877	4.5	2.6	3.6
CG32335	DRSC07948	4.5	2.6	3.6
rab3-GEF	DRSC19956	3.5		3.5
hook	DRSC18674	3.5		3.5
l(1)10Bb	DRSC20346	3.8	3.1	3.5
RpL13A	DRSC12265	3.5	3.5	3.5
E2f	DRSC16655	4.1	2.9	3.5
E23	DRSC00425	4.0	2.9	3.5
Pabp2	DRSC07501	2.5	4.4	3.5
CG9164	DRSC19087	3.2	3.7	3.5
CG34422	DRSC20029	3.2	3.7	3.5
Camta	DRSC06441	4.2	2.6	3.4
CG10927	DRSC06014	3.2	3.6	3.4
RpS23	DRSC07169	3.3	3.5	3.4
fru	DRSC16951	3.4		3.4
RpL37A	DRSC02899	4.2	2.6	3.4
luna	DRSC06663	3.7	3.1	3.4
scruff	DRSC12604	4.8	2.0	3.4
CG31179	DRSC13494	3.4		3.4
CG12470	DRSC18620	3.4		3.4
CG42389	DRSC02245	3.9	2.9	3.4
CG12484	DRSC05883	3.0	3.8	3.4
CG32062	DRSC10157	5.0	1.8	3.4
RpS27A	DRSC03421	3.0	3.7	3.4
Proctolin	DRSC03009	5.1	1.6	3.4
CG9253	DRSC03171	4.2	2.5	3.4

RpS27	DRSC14244	5.1	1.6	3.4
A3-3	DRSC17225	3.5	3.2	3.4
HDC10243 ('+' in Hild et al)	DRSC09354	3.2	3.5	3.4
RpII33	DRSC03415	3.0	3.6	3.3
homeobrain	DRSC04082	4.0	2.6	3.3
CG30460	DRSC06560	4.2	2.4	3.3
CG17329	DRSC01926	3.3		3.3
Furin 1	DRSC13707	3.2	3.4	3.3
Tektin-C	DRSC09741	3.0	3.6	3.3
HDC09523 ('+' in Stolc / Hild et al)	DRSC09076	4.5	2.1	3.3
CG4570	DRSC15602	4.1	2.4	3.3
Alhambra	DRSC12447	3.3		3.3
CG9007	DRSC11051	3.8	2.7	3.3
Snr1	DRSC12369	3.2	3.3	3.3
noisette	DRSC12383	3.2	3.3	3.3
CG32776	DRSC18119	4.6	1.9	3.3
rutabaga	DRSC20367	4.4	2.1	3.3
Sox100B	DRSC15090	3.2		3.2
Rrp45	DRSC20198	3.2		3.2
CG11138	DRSC19385	3.2		3.2
Cdc27	DRSC11112	2.8	3.6	3.2
mei-P26	DRSC17735	3.8	2.6	3.2
Hey	DRSC07440	5.2	1.2	3.2
CG32306	DRSC08333	4.2	2.2	3.2
msopa	DRSC11895	4.5	1.9	3.2
RpL6	DRSC14323	4.2	2.2	3.2
HDC17386	DRSC17342	3.4	3	3.2
bhringi	DRSC11000	3.1	3.3	3.2
CG3436	DRSC00605	4.2	2.1	3.2
CG3983	DRSC15529	5.0	1.3	3.2
shrub	DRSC07061	3.4	2.9	3.2
RpII140	DRSC16831	3.3	3	3.2
canoe	DRSC12374	4.6	1.7	3.2
thisbe	DRSC06153	4.6	1.7	3.2
alphaTub85E	DRSC16899	3.5	2.8	3.2
HDC08349 ('+' in Hild et al)	DRSC08080	4.2	2.1	3.2
HDC09253 ('+' in Hild et al)	DRSC08978	2.8	3.5	3.2
CG31461	DRSC13113	2.5	3.8	3.2
CG14543	DRSC14887	3.1		3.1
RnrL	DRSC03413	2.7	3.5	3.1
antimeros	DRSC12310	3.8	2.4	3.1
CG6937	DRSC16140	3.4	2.8	3.1
Rpb7	DRSC16068	2.7	3.5	3.1

CG31847	DRSC02535	2.9	3.3	3.1
Rgk1	DRSC07344	2.7	3.5	3.1
HDC13476 ('+' in Hild et al)	DRSC12996	3.2	3	3.1
D19A	DRSC11133	3.5	2.6	3.1
U2af38	DRSC00796	3.2	2.9	3.1
cropped	DRSC03515	2.7	3.4	3.1
Mrtf	DRSC08263	4.1	2.0	3.1
HDC07103 ('+' in Hild et al)	DRSC05711	4.2	1.9	3.1
CG31353	DRSC13530	5.8	0.3	3.1
translucent	DRSC11308	4.5	1.5	3.0
CG2446	DRSC19847	3.0		3.0
CG2685	DRSC18463	3.0		3.0
CG9777	DRSC20208	3.0		3.0
Rbm13	DRSC17749	3.0		3.0
Cbp20	DRSC16601	3.0		3.0
snRNP2	DRSC12536	2.8	3.2	3.0
His2B:CG17949	DRSC03757	3.3	2.7	3.0
HDC19527 ('+' in Hild et al)	DRSC19637	4.0	2.0	3.0
CG15630	DRSC00465	3.7	2.2	3.0
l(2)35Df	DRSC03560	3.6	2.3	3.0
roadkill	DRSC14526	3.4	2.5	3.0
CG31475	DRSC14762	3.4	2.5	3.0
Clipper	DRSC00746	2.9	3	3.0
CG10754	DRSC09801	2.7	3.2	3.0
His-Psi:CR31615/CR31616	DRSC03760	3.3	2.6	3.0
HDC05639 ('+' in Hild et al)	DRSC05231	3.1	2.8	3.0
HDC07480 ('+' in Stolc / Hild et al)	DRSC05871	5.2	0.7	3.0
Sec61alpha	DRSC03256	3.7	2.1	2.9
Spt5	DRSC07556	3.6	2.2	2.9
TweedleN	DRSC15813	2.9		2.9
CG4218	DRSC01939	2.9		2.9
HLH106	DRSC11182	3.5	2.3	2.9
CG15470	DRSC18053	4.1	1.7	2.9
HDC13798	DRSC13109	3.1	2.7	2.9
RpS11	DRSC07289	3.8	2.0	2.9
sine oculis	DRSC07693	3.1	2.7	2.9
Ef1alpha100E	DRSC16658	2.9		2.9
CG32970	DRSC01375	2.9		2.9
Fasciclin 2	DRSC17308	2.9		2.9
CG12278	DRSC14494	2.9		2.9
Cdc42	DRSC20228	3.3	2.4	2.9
CG6509	DRSC02964	2.8	2.9	2.9
Nup98	DRSC14209	3.6	2.0	2.8

peanuts	DRSC07120	3.2	2.4	2.8
CG32043	DRSC10330	4.3	1.3	2.8
bluecheese	DRSC02333	3.2	2.4	2.8
Dim1	DRSC00563	3.0	2.6	2.8
CG3563	DRSC13053	2.9	2.7	2.8
beat-IIb	DRSC13219	2.8		2.8
MED28	DRSC15721	2.8		2.8
couch potato	DRSC14503	2.8		2.8
anterior open	DRSC00801	4.6	0.9	2.8
Cpr66D	DRSC10646	3.9	1.6	2.8
CG9426	DRSC03219	3.6	1.9	2.8
MBD-R2	DRSC14180	3.1	2.4	2.8
Beadex	DRSC19350	2.6	2.9	2.8
CSN3	DRSC11859	3.4	2.0	2.7
CG31145	DRSC14670	4.6	0.8	2.7
Tim9b	DRSC19566	3.1	2.3	2.7
MED22	DRSC18175	2.7		2.7
CG15365	DRSC18038	4.0	1.4	2.7
Tim17b2	DRSC03457	2.7		2.7
HDC17481 ('+' in Hild et al)	DRSC17379	3.9	1.5	2.7
Neprilysin 1	DRSC18389	2.7		2.7
warts	DRSC17096	4.3	1.0	2.7
NF-YC-like	DRSC18272	3.9	1.4	2.7
HDC18875 ('+' in Hild et al)	DRSC19029	3.2	2.1	2.7
CG31229	DRSC13309	2.9	2.2	2.6
dalao	DRSC18419	2.9	2.2	2.6
Smrter	DRSC19495	3.8	1.4	2.6
CG18375	DRSC04305	2.7	2.5	2.6
CG4959	DRSC01938	2.6		2.6
CG5732	DRSC15863	2.6		2.6
CG30127	DRSC05786	2.6		2.6
vrille	DRSC03633	4.6	0.6	2.6
mRNA-capping-enzyme	DRSC19789	3.2	2.0	2.6
HDC08744 ('+' in Hild et al)	DRSC08800	3.8	1.4	2.6
Symplekin	DRSC12301	2.8	2.4	2.6
foxo	DRSC13017	3.2	2.0	2.6
HDC17467 ('+' in Hild et al)	DRSC17370	3.1	2.1	2.6
Rpb8	DRSC11642	2.7	2.4	2.6
CG3918	DRSC18331	3.7	1.4	2.6
CG5739	DRSC02885	3.1	2.0	2.6
CG4830	DRSC15657	3.1	2.0	2.6
CG12454	DRSC19458	3.7	1.4	2.6
HDC05227 ('+' in Hild et al)	DRSC05108	2.5	2.6	2.6

HDC08028	DRSC07960	2.9	2.2	2.6
spn-F	DRSC14475	2.8	2.3	2.6
Rbp2	DRSC20276	2.9	2.2	2.6
cut	DRSC18757	2.8	2.1	2.5
l(2)k09022	DRSC02108	3.8	1.2	2.5
CG14023	DRSC02353	2.9	2.1	2.5
Su(Tpl)	DRSC10954	2.8	2.2	2.5
CG8885	DRSC03117	2.8	2.2	2.5
CG13278	DRSC02252	2.5		2.5
CG7532	DRSC01910	2.5		2.5
Su(z)2	DRSC07558	3.0	2.0	2.5
eyegone	DRSC11344	3.3	1.7	2.5
HDC14033	DRSC13200	2.7	2.3	2.5
Hsp83	DRSC08664	2.7	2.2	2.5
CG7597	DRSC11836	2.5	2.4	2.5
CG2865	DRSC18528	2.8	2.1	2.5
smG	DRSC20207	2.9	2.0	2.5
HDC01635	DRSC01082	2.8	2.1	2.5
HDC10238	DRSC09350	2.6	2.3	2.5
HDC12272	DRSC12455	2.6	2.3	2.5
Stam	DRSC03444	2.6	2.2	2.4
pUf68	DRSC08731	3.4	1.4	2.4
CG12054	DRSC14467	2.6	2.2	2.4
Tor	DRSC02811	2.8	2.0	2.4
serpent	DRSC17068	2.7	2.1	2.4
CG4615	DRSC18376	3.0	1.7	2.4
CG5466	DRSC15808	3.7	1.1	2.4
CG15740	DRSC19706	2.8	2.0	2.4
dpr6	DRSC09005	3.9	0.9	2.4
Hsp70Ab	DRSC15380	2.9	1.8	2.4
hyrax	DRSC14462	2.7	2.0	2.4
CG5844	DRSC15890	2.5	2.2	2.4
HDC02165 ('+' in Hild et al)	DRSC01243	3.0	1.7	2.4
HDC02637 ('+' in Hild et al)	DRSC01452	2.7	2.0	2.4
HDC04273 ('+' in Stolc / Hild et al)	DRSC03998	2.7	2.0	2.4
HDC13786	DRSC13099	2.5	2.2	2.4
Fatty acid synthetase	DRSC00268	2.6	2.0	2.3
Neurocalcin	DRSC07022	2.5	2.1	2.3
PDK1	DRSC08682	3.0	1.5	2.3
Eip74EF	DRSC10613	3.3	1.3	2.3
HDC15185	DRSC14674	5.2	-0.6	2.3
CG33110	DRSC14694	3.5	1.1	2.3
HDC14620 ('+' in Stolc / Hild et al)	DRSC13410	5.0	-0.4	2.3

CG12650	DRSC17609	2.8	1.8	2.3
HDC08749 ('+' in Hild et al)	DRSC08805	2.8	1.8	2.3
CG31705	DRSC01991	2.9	1.6	2.3
bunched	DRSC03500	2.7	1.8	2.3
Klp61F	DRSC08671	2.7	1.8	2.3
pgant6	DRSC08552	2.7	1.8	2.3
CSN7	DRSC06807	2.5	2.0	2.3
CG15753	DRSC19718	4.6	-0.1	2.3
CG42270	DRSC19967	2.8	1.7	2.3
HDC09080 ('+' in Hild et al)	DRSC08919	5.6	-1.1	2.3
HDC18214 ('+' in Hild et al)	DRSC17632	2.7	1.8	2.3
dre4	DRSC08714	3.7	0.7	2.2
CG13779	DRSC02282	3.1	1.3	2.2
CG6043	DRSC02922	3.5	0.9	2.2
CG34113	DRSC12263	3.1	1.3	2.2
Hexokinase C	DRSC07079	2.8	1.6	2.2
TORC	DRSC10578	4.1	0.3	2.2
alphaTub84B	DRSC12622	2.8	1.6	2.2
CG6015	DRSC15948	2.7	1.7	2.2
CG34354	DRSC14558	4.3	0.1	2.2
Hsp70Bb	DRSC21248	2.7	1.7	2.2
HDC05820 ('+' in Stolc / Hild et al)	DRSC05297	2.8	1.6	2.2
snRNA:U1:95Cc	DRSC13640	4.2	0.2	2.2
Cct5	DRSC07357	3.5	0.8	2.2
muscleblind	DRSC07651	2.9	1.4	2.2
alphaTub84D	DRSC12623	3.5	0.8	2.2
HDC18498 ('+' in Stolc / Hild et al)	DRSC19661	3.7	0.6	2.2
araucan	DRSC11322	3.0	1.2	2.1
CG30387	DRSC04256	3.3	0.8	2.1
Mlf	DRSC07472	3.4	0.7	2.1
CG14982	DRSC08440	3.2	0.9	2.1
Tie	DRSC08702	2.8	1.3	2.1
CG3982	DRSC10394	2.8	1.3	2.1
HDC18670 ('+' in Hild et al)	DRSC18963	3.2	0.9	2.1
CG5446	DRSC02862	3.0	1.0	2.0
hook-like	DRSC02062	2.8	1.2	2.0
bubblegum	DRSC03495	3.2	0.7	2.0
Tbp	DRSC04662	2.6	1.4	2.0
CG17622	DRSC15247	3.9	0.1	2.0
HDC01575 ('+' in Hild et al)	DRSC01059	2.9	1.1	2.0
par-1	DRSC05792	2.7	1.3	2.0
HDC09511 ('+' in Stolc / Hild et al)	DRSC09067	4.4	-0.4	2.0
CG9817	DRSC18446	3.7	0.3	2.0

CG6854	DRSC10729	2.7	1.2	2.0
cryptocephal	DRSC03513	2.9	1.0	2.0
CG33558	DRSC05013	2.5	1.4	2.0
sticks and stones	DRSC06423	3.3	0.6	2.0
HDC11218 ('+' in Hild et al)	DRSC11443	2.6	1.3	2.0
HDC12421 ('+' in Hild et al)	DRSC12666	3.8	0.1	2.0
Nnp-1	DRSC02165	3.7	0.1	1.9
HDC09529 ('+' in Hild et al)	DRSC09082	2.9	0.9	1.9
HDC14879 ('+' in Hild et al)	DRSC13509	3.7	0.1	1.9
CG30497	DRSC06425	2.7	1.0	1.9
Gbp	DRSC07434	2.5	1.2	1.9
CG32245	DRSC08327	3.8	-0.1	1.9
PP2A-B'	DRSC16337	3.0	0.7	1.9
HDC07450	DRSC05865	2.5	1.2	1.9
CG33980	DRSC18089	2.9	0.8	1.9
HDC18410 ('+' in Stolc / Hild et al)	DRSC18886	2.9	0.8	1.9
CG30349	DRSC06421	3.5	0.1	1.8
CG5151	DRSC10487	3.4	0.2	1.8
E5	DRSC16585	2.7	0.9	1.8
CG34436	DRSC15165	4.1	-0.5	1.8
Rtc1	DRSC20285	3.0	0.5	1.8
HDC14311 ('+' in Hild et al)	DRSC14786	3.5	0.0	1.8
CG6962	DRSC16153	2.7	0.7	1.7
CG4585	DRSC04475	3.1	0.3	1.7
CG11583	DRSC08202	2.8	0.6	1.7
fizzy	DRSC03534	2.7	0.7	1.7
CG6764	DRSC16111	2.9	0.5	1.7
Ten-a	DRSC19496	2.7	0.7	1.7
Vps4	DRSC19338	2.5	0.8	1.7
CG9836	DRSC16572	2.6	0.7	1.7
CG7031	DRSC16178	3.1	0.2	1.7
CG12071	DRSC14471	2.8	0.5	1.7
Pdp1	DRSC08896	3.7	-0.4	1.7
CG5525	DRSC02865	2.6	0.6	1.6
CG18131	DRSC00517	2.5	0.7	1.6
nop5	DRSC03582	2.6	0.6	1.6
CG14107	DRSC10110	3.2	-0.1	1.6
CG32432	DRSC11703	3.1	0.1	1.6
fd3F	DRSC17843	3.5	-0.3	1.6
HDC14836 ('+' in Hild et al)	DRSC13493	3.1	0.1	1.6
CG9300	DRSC11064	2.9	0.2	1.6
CG30349	DRSC06839	2.9	0.2	1.6
Mystery 45A	DRSC07067	2.6	0.5	1.6

Su(var)205	DRSC03446	2.6	0.4	1.5
HDC08645 ('+' in Stolc / Hild et al)	DRSC08772	2.9	0.1	1.5
HDC14699 ('+' in Stolc / Hild et al)	DRSC13445	3.4	-0.4	1.5
CG42337	DRSC11687	2.6	0.2	1.4
HDC14817 ('+' in Hild et al)	DRSC13481	2.8	0.0	1.4
pasilla	DRSC16388	2.6	0.1	1.4
HDC12808	DRSC12769	2.6	0.0	1.3
HDC15101 ('+' in Stolc / Hild et al)	DRSC13589	2.6	0.0	1.3
CG9948	DRSC09690	2.7	-0.2	1.3
CG12880	DRSC14568	2.6	-0.2	1.2
HDC08562	DRSC08743	2.7	-0.3	1.2
HDC11852 ('+' in Hild et al)	DRSC12050	2.6	-0.2	1.2
HDC14962 ('+' in Hild et al)	DRSC13553	2.7	-0.4	1.2
HDC02648 ('+' in Stolc / Hild et al)	DRSC01457	2.6	-0.4	1.1
HDC14638	DRSC13421	2.5	-0.5	1.0
