# Supplementary material for: Drosophila Genome-Wide RNAi Screen Identifies Multiple Regulators of HIF–Dependent Transcription in Hypoxia
Source: PLoS Genet. 2010 Jun 24;6(6):e1000994. doi: 10.1371/journal.pgen.1000994 (PMC2891703; doi:10.1371/journal.pgen.1000994)
Supplement: Table S3 — The data obtained at the primary screen (Table S2) were filtered against the results of a cell viability screen previously carried out at the DRSC (Boutros et al. 2004) [23]. Sequences from the “Sanger collection” were also eliminated from the study; the 225 genes that remained as positive hits of the primary screen are depicted. (0.27 MB PDF) [file pgen.1000994.s006.pdf]

Table S3. Filtered results of the primary screen.

Primary screen (filtered)				
Gene	Amplicon	-Z1	-Z2	Average -Z
no hitter	DRSC01976	9.3		9.3
brahma	DRSC11330	7.8	9.1	8.5
CG10660	DRSC09769	7.3	8.6	8.0
cyclophilin-33	DRSC07600	6.3	9.3	7.8
CG4174	DRSC10406	7.7		7.7
CG4587	DRSC01956	7.4		7.4
CG11985	DRSC14460	5.8	8.0	6.9
CG9119	DRSC08620	6.5	7.2	6.9
CG15278	DRSC02481	6.5		6.5
CG32000	DRSC17103	5.7	7.2	6.5
sima	DRSC17055	7.0	5.8	6.4
CG9134	DRSC07954	6.3		6.3
Trip1	DRSC03464	6.1	6.5	6.3
CG3605	DRSC00619	5.8	6.6	6.2
CG18591	DRSC02680	5.6	6.8	6.2
pixie	DRSC10533	5.7	6.4	6.1
CG8636	DRSC18427	5.5	6.4	6.0
Hsc70-4	DRSC16711	5.9	5.9	5.9
Ef2b	DRSC03737	5.2	6.5	5.9
CG15494	DRSC02502	5.7		5.7
SmB	DRSC03437	5.9	5.3	5.6
CG9769	DRSC12328	5.4	5.1	5.3
moira	DRSC15378	5.2	5.3	5.3
p130CAS	DRSC08257	5.5	4.9	5.2
NSL1	DRSC15625	4.8	5.6	5.2
Helicase at 25E	DRSC03342	5.1	5.1	5.1
CG15097	DRSC06526	5.1	5.0	5.1
ran	DRSC20364	4.2	5.8	5.0
DebB	DRSC07397	5.0	4.8	4.9
Rrp6	DRSC16223	4.2	5.6	4.9
CG5931	DRSC10559	4.1	5.6	4.9
SmD3	DRSC07553	3.9	5.8	4.9
squid	DRSC17066	4.8		4.8
raptor	DRSC18359	4.7		4.7

CG42342	DRSC14378	4.2	5.2	4.7
prp8	DRSC07293	5.3	4.1	4.7
CG7065	DRSC18420	3.6	5.7	4.7
Tango7	DRSC07142	5.2	3.8	4.5
CG5482	DRSC06936	6.1	2.8	4.5
CG8929	DRSC07298	3.8	5.1	4.5
CG4849	DRSC15662	5.0	3.8	4.4
CG17304	DRSC15222	4.7	4.1	4.4
CG15450	DRSC20522	4.6	4.2	4.4
Spt6	DRSC18836	4.0	4.8	4.4
AGO1	DRSC05912	6.1	2.6	4.4
chinmo	DRSC00509	3.6	5.1	4.4
SF2	DRSC16845	3.2	5.4	4.3
CSN6	DRSC16593	4.3		4.3
Jon99Ciii	DRSC16859	4.3		4.3
AdoR	DRSC16556	4.3		4.3
crooked-neck	DRSC18755	3.9	4.7	4.3
Patj	DRSC08712	4.0	4.5	4.3
Suv4-20	DRSC18482	3.9	4.6	4.3
snRNP69D	DRSC09800	5.7	2.7	4.2
TER94	DRSC07560	4.5	3.9	4.2
pontin	DRSC17029	4.5	3.9	4.2
ascutex	DRSC05924	2.7	5.7	4.2
reptin	DRSC11388	5.1	3.2	4.2
small bristles	DRSC20368	4.2	4.1	4.2
CG7757	DRSC10912	5.1	3.2	4.2
tango	DRSC17077	3.3	4.8	4.1
Bap60	DRSC19337	3.8	4.4	4.1
Slu7	DRSC14729	3.7	4.5	4.1
Tango4	DRSC19786	3.9	4.3	4.1
CG13298	DRSC09972	3.3	4.9	4.1
CG7918	DRSC16345	5.9	2.2	4.1
Trn-SR	DRSC00546	3.7	4.4	4.1
Su(var)3-9	DRSC13081	4.0	4.1	4.1
ph-d	DRSC18819	4.0		4.0
CG14641	DRSC12227	3.1	4.9	4.0
CG14180	DRSC10179	4.0	3.9	4.0
CG6197	DRSC06967	4.1	3.8	4.0
Pvf2	DRSC00968	2.6	5.2	3.9
Calx	DRSC13457	3.9		3.9
Neosin	DRSC11235	3.5	4.3	3.9
Ef1alpha48D	DRSC07421	3.5	4.3	3.9
Rheb	DRSC12148	3.0	4.7	3.9
CG14995	DRSC08452	4.6	3.1	3.9
CG12499	DRSC14521	3.8		3.8
Chrac-16	DRSC20343	3.8		3.8
U2af50	DRSC20297	3.5	4.0	3.8
bric a brac 1	DRSC08379	3.0	4.4	3.7
minibrain	DRSC20058	2.7	4.7	3.7
Ef1gamma	DRSC16659	2.8	4.5	3.7
l(3)03670	DRSC16981	3.6		3.6

stumps	DRSC15332	5.3	1.9	3.6
Rtf1	DRSC04085	3.8	3.4	3.6
Tcp1-like	DRSC16877	4.5	2.6	3.6
CG32335	DRSC07948	4.5	2.6	3.6
rab3-GEF	DRSC19956	3.5		3.5
hook	DRSC18674	3.5		3.5
l(1)10Bb	DRSC20346	3.8	3.1	3.5
CG9164	DRSC19087	3.2	3.7	3.5
CG34422	DRSC20029	3.2	3.7	3.5
Camta	DRSC06441	4.2	2.6	3.4
CG10927	DRSC06014	3.2	3.6	3.4
fruitless	DRSC16951	3.4		3.4
CG31179	DRSC13494	3.4		3.4
CG12470	DRSC18620	3.4		3.4
CG12484	DRSC05883	3.0	3.8	3.4
CG9253	DRSC03171	4.2	2.5	3.4
CG17329	DRSC01926	3.3		3.3
Furin 1	DRSC13707	3.2	3.4	3.3
Tektin-C	DRSC09741	3.0	3.6	3.3
CG4570	DRSC15602	4.1	2.4	3.3
Snr1	DRSC12369	3.2	3.3	3.3
Sox100B	DRSC15090	3.2		3.2
Rrp45	DRSC20198	3.2		3.2
CG11138	DRSC19385	3.2		3.2
bhringi	DRSC11000	3.1	3.3	3.2
CG3436	DRSC00605	4.2	2.1	3.2
CG3983	DRSC15529	5.0	1.3	3.2
thisbe	DRSC06153	4.6	1.7	3.2
CG31461	DRSC13113	2.5	3.8	3.2
CG14543	DRSC14887	3.1		3.1
RnrL	DRSC03413	2.7	3.5	3.1
CG6937	DRSC16140	3.4	2.8	3.1
CG31847	DRSC02535	2.9	3.3	3.1
D19A	DRSC11133	3.5	2.6	3.1
U2af38	DRSC00796	3.2	2.9	3.1
cropped	DRSC03515	2.7	3.4	3.1
CG2446	DRSC19847	3.0		3.0
CG2685	DRSC18463	3.0		3.0
CG9777	DRSC20208	3.0		3.0
Rbm13	DRSC17749	3.0		3.0
Cbp20	DRSC16601	3.0		3.0
snRNP2	DRSC12536	2.8	3.2	3.0
CG15630	DRSC00465	3.7	2.2	3.0
l(2)35Df	DRSC03560	3.6	2.3	3.0
roadkill	DRSC14526	3.4	2.5	3.0
Clipper	DRSC00746	2.9	3.0	3.0
CG10754	DRSC09801	2.7	3.2	3.0
Sec61alpha	DRSC03256	3.7	2.1	2.9
Spt5	DRSC07556	3.6	2.2	2.9
TweedleN	DRSC15813	2.9		2.9
CG4218	DRSC01939	2.9		2.9

Ef1alpha100E	DRSC16658	2.9		2.9
Fasciclin 2	DRSC17308	2.9		2.9
CG12278	DRSC14494	2.9		2.9
Cdc42	DRSC20228	3.3	2.4	2.9
CG6509	DRSC02964	2.8	2.9	2.9
Nup98	DRSC14209	3.6	2.0	2.8
peanuts	DRSC07120	3.2	2.4	2.8
blue cheese	DRSC02333	3.2	2.4	2.8
Dim1	DRSC00563	3.0	2.6	2.8
beat-IIb	DRSC13219	2.8		2.8
MED28	DRSC15721	2.8		2.8
Cpr66D	DRSC10646	3.9	1.6	2.8
CG9426	DRSC03219	3.6	1.9	2.8
MBD-R2	DRSC14180	3.1	2.4	2.8
Beadex	DRSC19350	2.6	2.9	2.8
CSN3	DRSC11859	3.4	2.0	2.7
Tim9b	DRSC19566	3.1	2.3	2.7
MED22	DRSC18175	2.7		2.7
Tim17b2	DRSC03457	2.7		2.7
Neprilysin 1	DRSC18389	2.7		2.7
CG31229	DRSC13309	2.9	2.2	2.6
dalao	DRSC18419	2.9	2.2	2.6
CG18375	DRSC04305	2.7	2.5	2.6
CG4959	DRSC01938	2.6		2.6
CG5732	DRSC15863	2.6		2.6
CG30127	DRSC05786	2.6		2.6
vrille	DRSC03633	4.6	0.6	2.6
Symplekin	DRSC12301	2.8	2.4	2.6
CG5739	DRSC02885	3.1	2.0	2.6
CG4830	DRSC15657	3.1	2.0	2.6
spn-F	DRSC14475	2.8	2.3	2.6
Rbp2	DRSC20276	2.9	2.2	2.6
cut	DRSC18757	2.8	2.1	2.5
l(2)k09022	DRSC02108	3.8	1.2	2.5
Su(Tpl)	DRSC10954	2.8	2.2	2.5
CG8885	DRSC03117	2.8	2.2	2.5
CG13278	DRSC02252	2.5		2.5
CG7532	DRSC01910	2.5		2.5
Hsp83	DRSC08664	2.7	2.2	2.5
CG7597	DRSC11836	2.5	2.4	2.5
CG2865	DRSC18528	2.8	2.1	2.5
SmG	DRSC20207	2.9	2.0	2.5
Stam	DRSC03444	2.6	2.2	2.4
pUf68	DRSC08731	3.4	1.4	2.4
Tor	DRSC02811	2.8	2.0	2.4
CG4615	DRSC18376	3.0	1.7	2.4
Hsp70Ab	DRSC15380	2.9	1.8	2.4
hyrax	DRSC14462	2.7	2.0	2.4
Fatty acid synthetase	DRSC00268	2.6	2.0	2.3
Neurocalcin	DRSC07022	2.5	2.1	2.3
PDK1	DRSC08682	3.0	1.5	2.3

CG31705	DRSC01991	2.9	1.6	2.3
bunched	DRSC03500	2.7	1.8	2.3
Klp61F	DRSC08671	2.7	1.8	2.3
pgant6	DRSC08552	2.7	1.8	2.3
CSN7	DRSC06807	2.5	2.0	2.3
dre4	DRSC08714	3.7	0.7	2.2
CG13779	DRSC02282	3.1	1.3	2.2
CG34113	DRSC12263	3.1	1.3	2.2
Hexokinase C	DRSC07079	2.8	1.6	2.2
CG6015	DRSC15948	2.7	1.7	2.2
Hsp70Bb	DRSC21248	2.7	1.7	2.2
Cct5	DRSC07357	3.5	0.8	2.2
muscleblind	DRSC07651	2.9	1.4	2.2
Mlf	DRSC07472	3.4	0.7	2.1
Tie	DRSC08702	2.8	1.3	2.1
CG5446	DRSC02862	3.0	1.0	2.0
hook-like	DRSC02062	2.8	1.2	2.0
bubblegum	DRSC03495	3.2	0.7	2.0
CG6854	DRSC10729	2.7	1.2	2.0
cryptocephal	DRSC03513	2.9	1.0	2.0
Nnp-1	DRSC02165	3.7	0.1	1.9
Gbp	DRSC07434	2.5	1.2	1.9
CG32245	DRSC08327	3.8	-0.1	1.9
CG30349	DRSC06421	3.5	0.1	1.8
Rtc1	DRSC20285	3.0	0.5	1.8
CG6962	DRSC16153	2.7	0.7	1.7
CG4585	DRSC04475	3.1	0.3	1.7
CG11583	DRSC08202	2.8	0.6	1.7
Vps4	DRSC19338	2.5	0.8	1.7
CG5525	DRSC02865	2.6	0.6	1.6
CG18131	DRSC00517	2.5	0.7	1.6
CG14107	DRSC10110	3.2	-0.1	1.6
CG9300	DRSC11064	2.9	0.2	1.6
Mystery 45A	DRSC07067	2.6	0.5	1.6
Su(var)205	DRSC03446	2.6	0.4	1.5
pasilla	DRSC16388	2.6	0.1	1.4
CG9948	DRSC09690	2.7	-0.2	1.3
CG12880	DRSC14568	2.6	-0.2	1.2
