# Supplementary material for: Drosophila Genome-Wide RNAi Screen Identifies Multiple Regulators of HIF–Dependent Transcription in Hypoxia
Source: PLoS Genet. 2010 Jun 24;6(6):e1000994. doi: 10.1371/journal.pgen.1000994 (PMC2891703; doi:10.1371/journal.pgen.1000994)
Supplement: Table S4 — The secondary screen was also carried out in cells exposed to DFO. A second-generation library (DRSC 2.0 library) was used, in which most genes are represented by more than one dsRNA. Normalized luciferase activity (firefly/renilla luciferase activity ratio) for each well was calculated and expressed as a percentage of the inhibition respect to control cells treated with dsRNA against GFP that were exposed to DFO. The screen was carried out in duplicate and the mean percentage of inhibition is depicted. (0.26 MB PDF) [file pgen.1000994.s007.pdf]

Table S4. Secondary screen.

Secondary screen				
**Gene**	**Amplicon 1**	**Inhibition(%)**	**Amplicon 2**	**Inhibition(%)**
**GROUP A: Inhibition of the DFO response > 75%**				
sima	DRSC33101	96,8 +/- 1,0	DRSC33100	96,4 +/- 0,5
tango	DRSC37262	95,9 +/- 0,6		
brahma	DRSC30902	89,0 +/- 4,1	DRSC30901	85,0 +/- 2,4
Trip1	DRSC32225	87,4 +/- 9,5	DRSC32224	87,3 +/- 0,7
CG14641	DRSC31837	85,7 +/- 0,2	DRSC31836	80,2 +/- 1,0
Ef2b	DRSC32107	85,6 +/- 2,7	DRSC32106	77,4 +/- 7,4
CG7065	DRSC35474	85,2 +/- 2,0		
tango7	DRSC31146	85,1 +/- 3,9	DRSC31145	86,0 +/- 0,0
Spt6	DRSC31075	83,4 +/- 0,0	DRSC31074	69,1 +/- 6,8
AGO1	DRSC30762	83,2 +/- 3,0	DRSC30761	79,7 +/- 2,8
pixie	DRSC32078	81,8 +/- 1,4	DRSC32077	80,6 +/- 7,4
moira	DRSC32754	78,6 +/- 3,9	DRSC32753	83,1 +/- 1,2
Snr1	DRSC32770	78,4 +/- 6,7	DRSC32769	56,4 +/- 5,1
Symplekin	DRSC32363	78,2 +/- 10,7	DRSC32362	81,9 +/- 5,8
CG4849	DRSC31791	76,5 +/- 17,3	DRSC31790	75,3 +/- 8,2
TER94	DRSC32221	75,9 +/- 3,2	DRSC32220	76,5 +/- 11,9
reptin	DRSC32532	75,5 +/- 15,2	DRSC32531	77,7 +/- 5,3
MBD-R2	DRSC32495	75,2 +/- 0,8	DRSC32494	73,4 +/- 7,9
NSL1	DRSC31789	74,5 +/- 2,8	DRSC31788	84,4 +/- 4,7
prp8	DRSC31801	74,3 +/- 2,8	DRSC31800	85,3 +/- 6,9
peanuts	DRSC31799	68,3 +/- 8,4	DRSC31798	80,9 +/- 6,9
CG9769	DRSC32096	66,7 +/- 9,3	DRSC32095	81,2 +/- 16,3
CG2446	DRSC32889	62,2 +/- 1,5	DRSC32888	81,0 +/- 1,3
**GROUP B: Inhibition of the DFO response 50 - 75%**				
Helicase at 25E	DRSC30680	72,7 +/- 3,0	DRSC30679	61,4 +/- 21,0
tango4	DRSC31783	70,8 +/- 3,4	DRSC31782	43,4 +/- 2,3
pontin	DRSC31863	70,7 +/- 2,2	DRSC31862	72,4 +/- 1,6

CG5931	DRSC32420	70,4 +/- 18,2	DRSC32419	58,8 +/- 1,8
CG32000	DRSC32056	70,2 +/- 12,7	DRSC32055	60,7 +/- 1,6
U2af38	DRSC28068	70,1 +/- 15,6		
CSN3	DRSC32998	69,4 +/- 8,6	DRSC32997	71,9 +/- 2,6
CSN6	DRSC35658	68,8 +/- 19,6		
cropped	DRSC30708	66,0 +/- 9,0		
CG9253	DRSC37278	65,8 +/- 7,1		
CG18591	DRSC31785	65,7 +/- 23,6	DRSC31784	22 +/- 28,7
CG14543	DRSC26647	64,1 +/- 4,5		
U2af50	DRSC37266	64,0 +/- 2,5		
CG7757	DRSC30869	63,1 +/- 18,6	DRSC30868	63,4 +/- 1,4
Gbp	DRSC34548	62,7 +/- 3,5	DRSC34547	51,0 +/- 2,5
dalao	DRSC35681	60,3 +/- 41,0		
crooked-neck	DRSC31853	60,0 +/- 41,1	DRSC31852	64,1 +/- 10,2
Rrp6	DRSC32601	59,5 +/- 41,2	DRSC32600	40,0 +/- 22,0
Tor	DRSC30664	59,4 +/- 41,3	DRSC30663	39,5 +/- 12,5
CG10754	DRSC33610	59,3 +/- 41,4		
CG11583	DRSC32312	58,5 +/- 41,5	DRSC32311	52,0 +/- 7,9
CG6509	DRSC37258	58,3 +/- 41,6		
Sec61alpha	DRSC31246	58,3 +/- 41,7	DRSC31245	12,4 +/- 3,8
lethal(1)10Bb	DRSC31944	58,1 +/- 41,8	DRSC31943	49,2 +/- 52,6
Su(var)3-9	DRSC27837	58,0 +/- 41,9		
CG6197	DRSC31706	57,5 +/- 41,10	DRSC31705	47,2 +/- 44,0
SmD3	DRSC32012	57,4 +/- 41,11		
Dim1	DRSC33266	56,6 +/- 41,12	DRSC33265	55,5 +/- 7,9
TweedleN	DRSC25037	56,4 +/- 41,13		
squid	DRSC32626	55,7 +/- 41,14	DRSC32625	22,3 +/- 26,9
Bap60	DRSC32657	55,5 +/- 41,15	DRSC32656	46,4 +/- 11,1
raptor	DRSC31050	55,2 +/- 41,16	DRSC31049	63,5 +/- 0,9
clipper	DRSC37267	54,1 +/- 41,17		
CG13779	DRSC30654	53,9 +/- 41,18	DRSC30653	70,2 +/- 9,2
lethal(2)35Df	DRSC32489	52,9 +/- 41,19	DRSC32488	38,9 +/- 22,5
Mlf	DRSC35853	52,6 +/- 41,20		
Rbp2	DRSC37291	50,5 +/- 41,21		
cryptocephal	DRSC37279	49,9 +/- 41,22		
Spt5	DRSC31820	48,1 +/- 41,23	DRSC31819	52,2 +/- 1,3
pUf68	DRSC31973	42,3 +/- 41,24	DRSC31972	62,2 +/- 16,8
Nup98	DRSC31804	40,9 +/- 41,25	DRSC31803	59,6 +/- 11,1
CG3605	DRSC31847	40,7 +/- 41,26	DRSC31846	57,8 +/- 15,6
PDK1	DRSC30826	36,1+/- 41,27	DRSC30825	51,0 +/- 29,6
**Inhibition of the DFO response < 50%**				
hyrax	DRSC30952	49,3 +/- 0,4	DRSC30951	33,0 +/- 24,6
Hsp83	DRSC33050	48,2 +/- 9,9	DRSC33049	45,4 +/- 12,5
Pvf2	DRSC35971	47,7 +/- 47,4		
SF2	DRSC32605	47,4 +/- 27,0	DRSC32604	51,8 +/- 4,1
CG5525	DRSC35379	47,0 +/- 17,0		
bhringi	DRSC32022	46,9 +/- 3,3	DRSC32021	20,6 +/- 13,8
Rtf1	DRSC30728	46,8 +/- 14,7	DRSC30727	37,5 +/- 8,5
DebB	DRSC31802	46,6 +/- 34,2		

CG11985	DRSC31771	45,5 +/- 1,0	DRSC31770	49,7 +/- 6,0
Slu7	DRSC31779	44,3 +/- 35,0	DRSC31778	23,6 +/- 61,8
p130CAS	DRSC34850	43,1 +/- 9,9		
Su(var)205	DRSC37257	42,3 +/- 10,8		
CG14180	DRSC32333	40,3 +/- 42,3	DRSC32332	20,3 +/- 19,1
bunched	DRSC32803	39,1 +/- 15,4		
SmG	DRSC35622	38,7 +/- 10,4	DRSC35621	37,4 +/- 3,9
CG15278	DRSC37276	38,1 +/- 6,2		
CG4959	DRSC37273	37,9 +/- 10,9		
Rheb	DRSC30920	37,8 +/- 9,8	DRSC30919	46,6 +/- 21,3
CG6962	DRSC32278	36,3 +/- 18,1	DRSC32277	16 +/- 30,2
CG31461	DRSC35138	36,2 +/- 16,3	DRSC35137	19,7 +/- 17,3
CG2685	DRSC32365	36,1 +/- 34,0	DRSC32364	45,4 +/- 52,8
Su(Tpl)	DRSC36037	34,2 +/- 15,5		
Tcp1-like	DRSC34599	33,1 +/- 9,3	DRSC34598	22,5 +/- 6,2
snRNP2	DRSC31833	32,7 +/- 0,7	DRSC31832	25,1 +/- 26,4
CG14995	DRSC31557	32,6 +/- 17,2		
cut	DRSC25100	31,2 +/- 14,6		
CG6015	DRSC32424	31,1 +/- 38,9	DRSC32423	43,3 +/- 20,8
CG9777	DRSC35623	30,5 +/- 0,3		
fruitless	DRSC31020	30,3 +/- 26,5		
CG8929	DRSC34413	30,1 +/- 29,5	DRSC34412	47,6 +/- 5,2
MED22	DRSC35840	29,7 +/- 30,2		
CG3983	DRSC37285	29,2 +/- 3,9		
Rtc1	DRSC35998	28,6 +/- 3,8		
CG9948	DRSC37281	28,6 +/- 9,7		
small bristles	DRSC33352	28,3 +/- 21,5	DRSC33351	32,4 +/- 7,2
CG18131	DRSC31565	28 +/- 15,4	DRSC31564	28,1 +/- 10,8
CG30349	DRSC33264	27,6 +/- 21,2	DRSC33263	22 +/- 9,7
CG14107	DRSC32857	27,3 +/- 16,8		
Neosin	DRSC35885	26,9 +/- 25,1		
Sox100B	DRSC32624	25,9 +/- 11,7		
Cdc42	DRSC31437	25,9 +/- 21,0	DRSC31436	29,1 +/- 29,5
Ef1alpha48D	DRSC32105	25,7 +/- 2,1		
CG14210	DRSC29021	25,4 +/- 25,4		
Rbm13	DRSC33595	25,3 +/- 3,9		
CG32245	DRSC31575	25,2 +/- 2,6	DRSC31574	29,8 +/- 24,0
dre4	DRSC33008	24,6 +/- 4,6	DRSC33007	14,8 +/- 2,5
bric a brac 1	DRSC32796	24,6 +/- 19,4		
CG34159	DRSC31584	24,5 +/- 6,9		
CG9300	DRSC32094	24,4 +/- 20,3	DRSC32093	7,2 +/- 25,6
rab3-GEF	DRSC31094	24,2 +/- 3,1	DRSC31093	44,2 +/- 0,0
SmB	DRSC31869	23,9 +/- 11,4	DRSC31868	18,7 +/- 36,2
CG31179	DRSC35106	23,7 +/- 9,1		
Hsp70Bb	DRSC34562	23,6 +/- 25,6		
Hsp70Ab	DRSC29327	23,3 +/- 35,3		
no hitter	DRSC35889	22,5 +/- 16,4		
CG3436	DRSC32274	22,5 +/- 1,5	DRSC32273	13,1 +/- 9,8
tektin-C	DRSC32219	22,3 +/- 22,1	DRSC32218	59,4 +/- 15,6
Cct5	DRSC32024	22,0 +/- 27,6	DRSC32023	25,1 +/- 10,1
CG31705	DRSC30648	21,5 +/- 9,4		

camta	DRSC26757	21,5 +/- 39,1		
Tie	DRSC30830	21,3 +/- 17,4	DRSC30829	42,3 +/- 8,9
lethal(2)k09022	DRSC32028	20,1 +/- 4,2	DRSC32027	18,4 +/- 11,2
CG6937	DRSC32426	19,6 +/- 3,4	DRSC32425	12,2 +/- 23,8
CG42243	DRSC25912	19,5 +/- 10,9		
stam	DRSC37259	19,4 +/- 13,7		
hook	DRSC31032	19,1 +/- 0,0	DRSC31031	11,6 +/- 0,0
CG17329	DRSC28365	18,8 +/- 16,1		
CG34113	DRSC24955	18,6 +/- 7,3		
CG4218	DRSC27814	18,3 +/- 5,6		
CG31847	DRSC35170	18,3 +/- 9,1		
furin 1	DRSC35749	18,2 +/- 29,9		
CG4174	DRSC37283	17,6 +/- 25,3		
CG13298	DRSC30848	17,3 +/- 9,5		
CG4587	DRSC37274	16,7 +/- 3,7		
CG12484	DRSC28353	16,6 +/- 9,3		
CG10660	DRSC33244	16,5 +/- 12,4		
CG13278	DRSC37275	16,0 +/- 6,3		
Ef1alpha100E	DRSC32104	15,6 +/- 25,0	DRSC32103	24,7 +/- 40,7
minibrain	DRSC24961	15,1 +/- 0,1		
CG15097	DRSC37260	14,7 +/- 9,5		
fasciclin 2	DRSC35731	13,4 +/- 20,7		
Rrp45	DRSC29319	12,5 +/- 6,9		
Mystery 45A	DRSC32156	12,4 +/- 22,2	DRSC32155	14,3 +/- 33,8
CG5732	DRSC31849	12,2 +/- 27,9	DRSC31848	12,2 +/- 16,3
Cbp20	DRSC32000	11,9 +/- 15,3	DRSC31999	15,3 +/- 8,2
CG32335	DRSC25511	10,8 +/- 28,2		
beadex	DRSC33491	10,6 +/- 14,2		
CG9164	DRSC37295	10,4 +/- 39,5		
CG9119	DRSC35587	10,3 +/- 26,8		
Tim9b	DRSC32272	10,1 +/- 50,9		
CG8636	DRSC32088	9,9 +/- 16,6	DRSC32087	90,4 +/- 0,7
calx	DRSC36151	9,3 +/- 14,2		
Ef1gamma	DRSC35716	9,0 +/- 5,0		
ph-d	DRSC32519	8,8 +/- 4,8	DRSC32518	6,2 +/- 7,0
CG9134	DRSC23750	8,6 +/- 23,3		
ascutex	DRSC31953	7,3 +/- 27,2	DRSC31952	6,2 +/- 10,4
lethal(3)03670	DRSC26451	7,3 +/- 1,7		
CG34422	DRSC35485	6,9 +/- 0,4		
CG5446	DRSC36259	6,5 +/- 2,9		
Klp61F	DRSC30824	6,2 +/- 60,4	DRSC30823	31,8 +/- 10,6
CG30127	DRSC37293	6,0 +/- 23,2		
CG4570	DRSC37284	5,0 +/- 5,6		
CG15450	DRSC37292	5,0 +/- 11,5		
